# Complexities of assessing palaeocave stratigraphy: reconstructing site formation of the ∼2.61 Ma Drimolen Makondo fossil site

**DOI:** 10.7717/peerj.10360

**Published:** 2020-12-21

**Authors:** Ashleigh Murszewski, Giovanni Boschian, Andy I.R. Herries

**Affiliations:** 1 The Australian Archaeomagnetism Laboratory, Department of Archaeology and History, La Trobe University, Bundoora, VIC, Australia; 2 School of Earth, Atmosphere and Environment, Monash University, Clayton, VIC, Australia; 3 Biology Department, University of Pisa, Pisa, Italy; 4 Palaeo-Research Institute, University of Johannesburg, Auckland Park, Gauteng, South Africa

**Keywords:** Micromorphology, Sedimentology, Pliocene, Early Pleistocene, Palaeocave

## Abstract

Palaeocave sites in South Africa are world renowned repositories for palaeontological and archaeological material, dating from the terminal Pliocene to the Early Pleistocene. Due to their antiquity, complex karstification history and multifaceted infilling phases, palaeocave sites are notoriously difficult to contextualise. Further to this, 19th century lime-mining and diverse excavation and sampling techniques, have complicated stratigraphic interpretations of fossil-bearing deposits within the region. Locating and assessing newly discovered, minimally disturbed palaeocave sites allow for contextual information to be gathered with greater confidence and can aid in constructing a more robust understanding of the South African fossil record. Here, we use Drimolen Makondo; a minimally lime-mined ∼2.61 Ma palaeontological site, to apply a series of in-depth stratigraphic and micromorphological studies. Contextual data presented within this study, testifies to a relatively rapid infill with greater fluvial activity when compared to adjacent deposits at the younger ∼2.04–1.95 Ma Drimolen Main Quarry. The quantity of articulated macromammalian remains, high density of micromammalian remains and pollen identified, also highlights Drimolen Makondo as a key site for ongoing palaeoenvironmental studies at the Pliocene to Pleistocene transition in South Africa.

## Introduction

**Figure 1:**
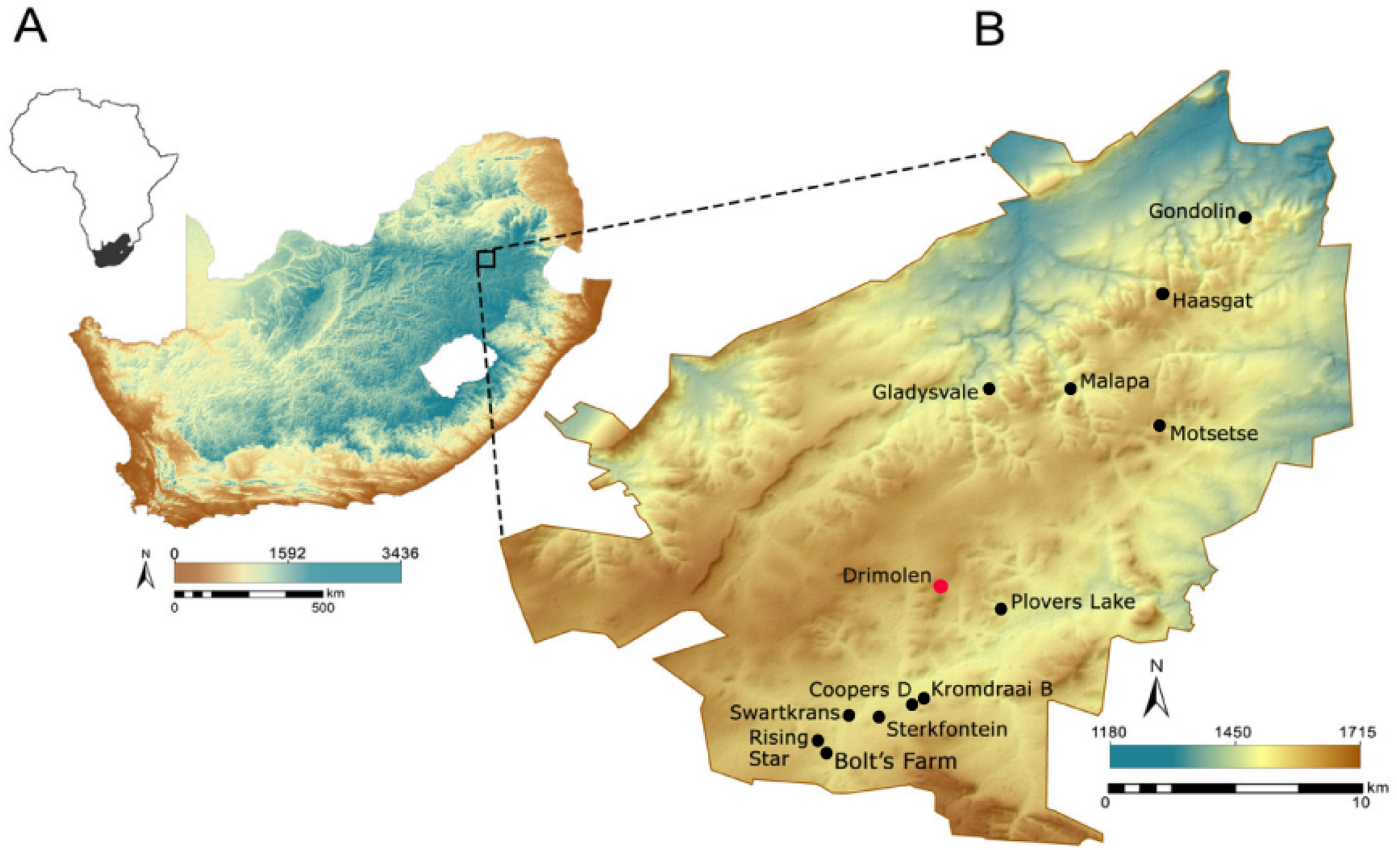
The location of the Drimolen palaeocave complex. (A) Topographic image showing the location of the Fossil Hominid Sites in South Africa. (B) Topographic images showing the location of sites (black dots) within the Fossil Hominid Sites of South Africa with their elevations. Red dot: Drimolen.

The Fossil Hominid Sites of South Africa ‘UNESCO World Heritage Area’ (referred to here as the Cradle of Humankind [alt. CoH]: Figs. 1A and 1B), is a geographical area that constrains a dense accumulation of palaeocave sites as old as the late Pliocene (Adams et al., 2010; Herries & Adams, 2013; Granger et al., 2015; Stratford et al., 2017). Palaeocaves are defined as features formed by agents active in the karst environment in the past (Bosák et al., 2015; pp. 25) that have been decoupled from active hydrogeochemical systems (Ford, 1995), infilled with relict sediments and are heavily eroded. A few palaeocave sites in South Africa are still associated with active cave systems and have been reworked within more recent karstification phases (Stratford & Palmer, 2017). Similar intersections of active cave passages and palaeokarst have been identified in Australia (Osborne, 1999; Osborne, 2007). For over a century, these karst landforms in South Africa have been a major source of information for palaeontology, palaeoanthropology and archaeology from the terminal Pliocene to Early Pleistocene (Broom, 1938; Dart, 1925). Specifically, South African palaeocaves serve as depositional repositories for some of the densest concentrations of early hominin remains in the world, including well preserved partial skeletons (Berger et al., 2010; Berger et al., 2015; Clarke, 2019; Keyser et al., 2000). Such deposits are palaeontological and archaeological data sources for several reasons: a collection of various carcasses within a ‘death trap’ accumulation (Val et al., 2015); a source of shelter or occupation for various species (e.g., baboon sleeping sites, porcupine accumulations (Brain, 1981; Bountalis & Kuhn, 2014)); or, as depositional repositories for fluvially transported material from the surrounding landscape (Adams et al., 2010; Berger et al., 2010; Berger et al., 2015; Caruana, 2017; Clarke, 2019; Herries & Adams, 2013; Herries et al., 2020; Granger et al., 2015; Murszewski et al., 2019; Stratford & Palmer, 2017; Stammers, Caruana & Herries, 2018).

**Figure 2:**
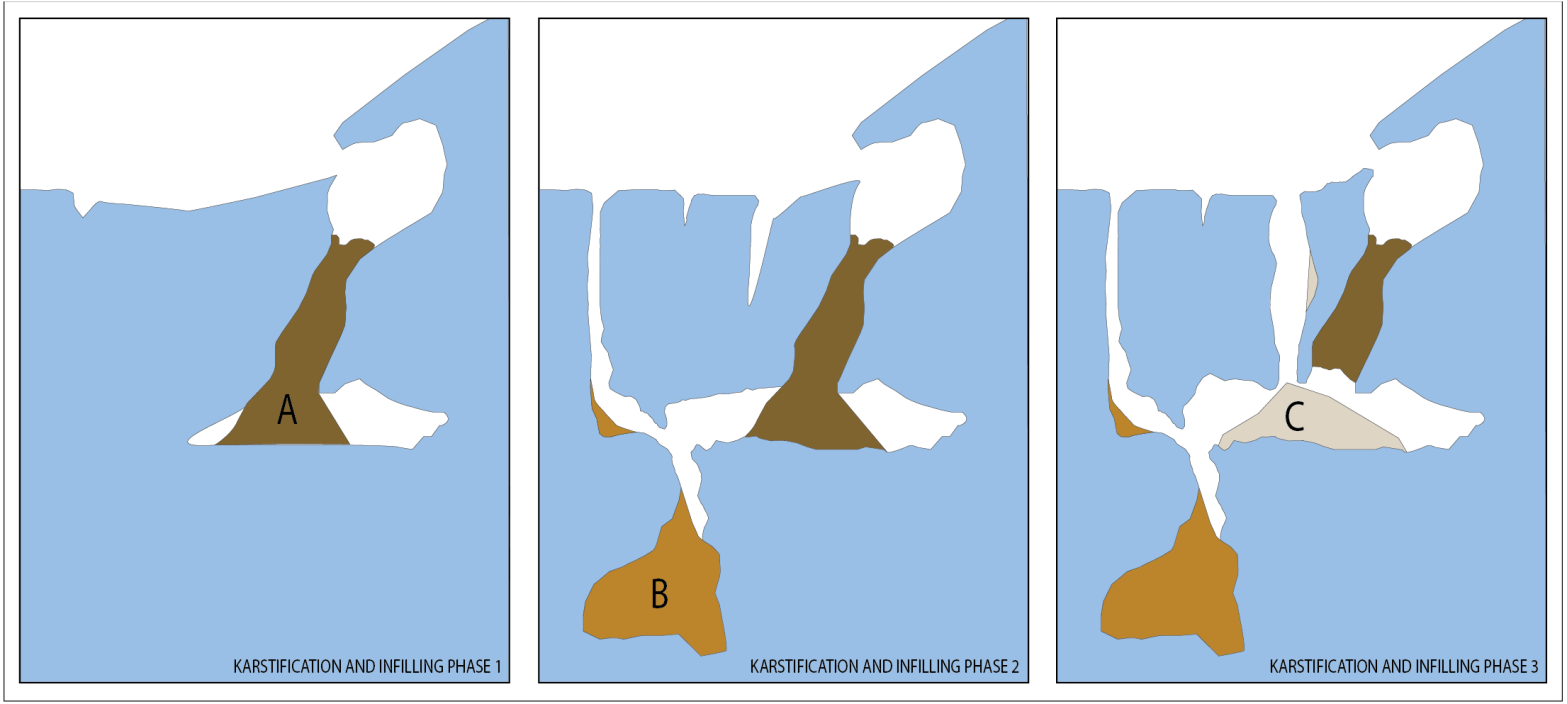
Schematic sketch of complex cave-infill systems with separate stages of karstification promoted through dissolution and erosion of the dolomite. Three separate phases are described. (A) Early collapse post small chamber formation during karstification and infilling phase 1. (B) Younger collapse in a separate chamber formed during karstification and infilling phase 2, which also resulted in the widening of the chamber of karstification and infilling phase 1. (C) Third phase of karstification and cave infill; resulting in more recent talus collapse mixed with oldest deposits from A.

Increase in the age of a karst landscape ultimately results in increasing complexity in the karst speleogenesis and subsequent infilling stages (Ford & Williams, 2013). As the host rock of palaeocave sites in CoH form one of the oldest karst landscapes in the world (Malmani Dolomite Succession, ∼2.6 Ga; Eriksson & Reczko, 1995; Murszewski et al., 2019), complex and multi-phased karstic systems are expected (Fig. 2). Palaeocave allogenic fill (inc. detrital silicates and often cemented by calcite) within the CoH is largely confined between ∼3 and ∼1 Ma (Herries & Adams, 2013; Herries & Shaw, 2011; Herries et al., 2009; Herries et al., 2013; Gibbon et al., 2014; Pickering & Kramers, 2010; Pickering et al., 2019; Schwartz, Ziari & Trivedi, 1994). However, age ranges of ∼4 Ma (Partridge et al., 2003) and ∼3.7 Ma (Granger et al., 2015) have been presented for the StW573 australopithecine fossil from Sterkfontein, though the latter has been more recently contested (2.8–2.2 Ma; Kramers & Dirks, 2017a; Kramers & Dirks, 2017b; Stratford & Palmer, 2017). A more recent archaeological record can also be found from ∼300 ka with the discovery of hominin remains such as *Homo naledi* at Rising Star (Dirks et al., 2017) and stone tools at sites such as Lincoln Cave at Sterkfontein (Reynolds et al., 2003; Reynolds, Clarke & Kuman, 2007).

**Figure 3:**
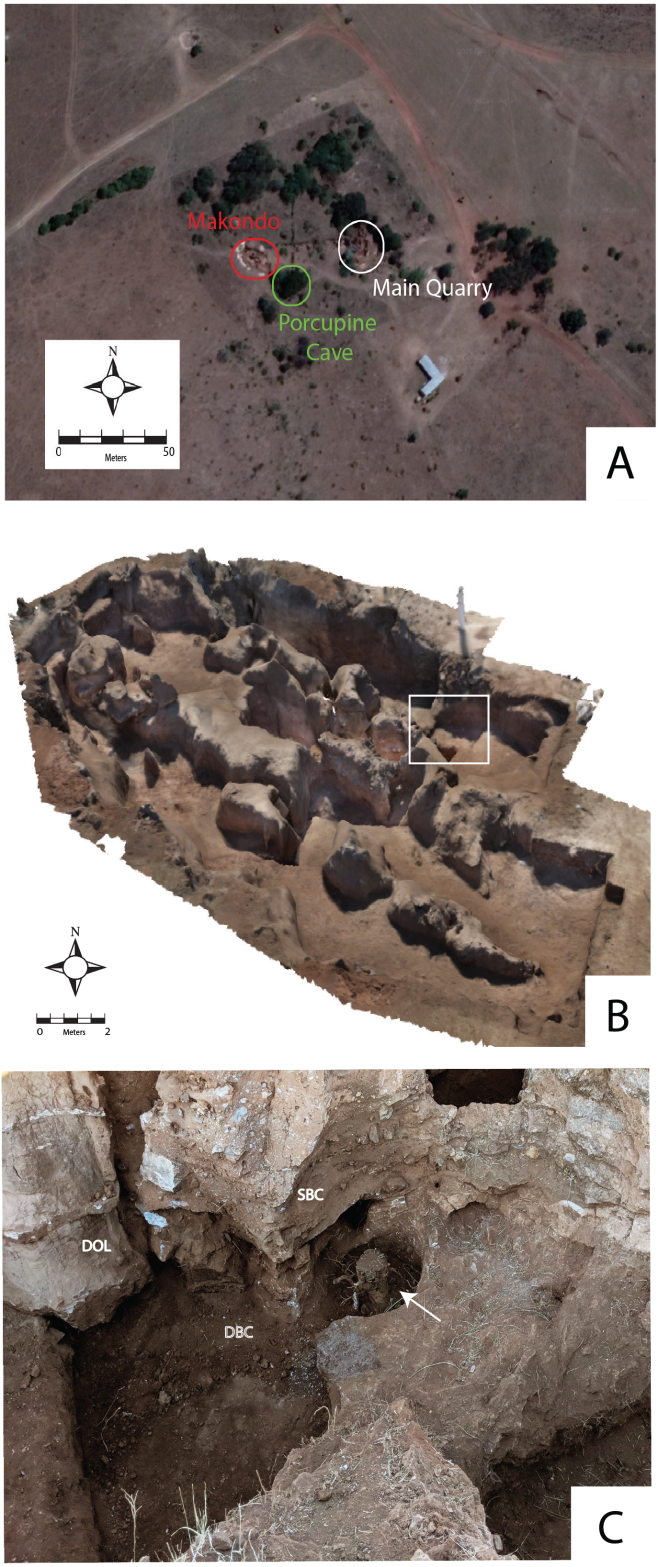
Drimolen Makondo. (A) Spatial proximity of three different sites at Drimolen: (1) Main Quarry, (2) Makondo and (3) Porcupine Cave. Aerial photograph from Google Maps. (B) 3D model of the Drimolen Makondo, showing the extents of the site. White box: location of C. (C) Image showing spatial relationship between makondo formation and vegetation growth (white arrow). SBC, Reworked talus cone breccia and fluvial deposits. DBC, Decalcified breccia. DOL, Dolomite.

These date ranges for fossil-bearing fill in the CoH has clear implications to the discussion on broader geological constraints and mechanisms of karstification and subsequent erosion, to which different theories have been presented. In the CoH, uplift and south-westward tilting in the Miocene facilitated river incision, and may have facilitated karstification (Partridge, 1973). Based on uplift, erosion and exposure, dates from 5–8 Ma have been proposed for the karstification in the CoH and ceiling breakdown in the late Pliocene (Martini et al., 2003). Alternatively, Dirks & Berger (2013) propose that caves within the Gauteng region formed simultaneously (∼4 Ma) and were exposed to the surface at different periods causing temporal variation in allogenic palaeocave fill. More recent revisions by Dirks et al. (2016) state that though dolomites were exposed in the Miocene, karstification and opening of caves did not occur until the Pleistocene. Herries et al. (2019) states that karstification in the region is more fluid and has occurred during multiple periods, with evidence of younger cave passages forming through palaeocave deposits. While this discussion has yet to be fully resolved, recent geological studies at Drimolen are supportive of a multi-karstification phase and multi-stage infill model, which has resulted in spatial and temporal distinct palaeocave fills at the site (Fig. 3A, Herries et al., 2020). At multiple sites, both palaeocaves and active caves occur in close proximity (i.e., Rising Star: Dirks et al., 2017, Drimolen: Herries et al., 2018; Herries et al., 2020 and Sterkfontein: Stratford, 2011; Wilkinson, 1985), or polycyclic karstic processes occurring within older caves (i.e., Warthog Cave at Drimolen: Herries et al., 2020; Gladysvale: Herries, 2003).

Despite the discovery of new sites in the CoH in the last 25 years, ‘classic’ Blaaubankspruit (alt. Blaauwbank or Bloubankspruit) Valley sites of Sterkfontein, Swartkrans and Kromdraai B have yielded the bulk of Early Pleistocene hominin remains from South Africa (Fig. 1B). All three have complex multiphase karstification histories with numerous phases of cave formation and infilling; recent near-surface karstification of solution features underneath colluvium and vegetation (i.e., formation of makondos: Brink & Partridge, 1980); as well as significant surface erosion (Dirks et al., 2016). Makondo formation results from chemical weathering through subsurface dissolution from humic acids (Brink & Partridge, 1980). Alternatively, extensive chemical weathering can be caused by carbon dioxide produced by vegetation metabolism, fluctuating pH and water content (Nicosia & Stoops, 2017, p. 375), as well as by root penetration. This process results in sub-cylindrical erosional features filled by decalcified soil, that penetrate the dolomite (Dubois et al., 2014), and the upper zones of palaeocave sediments (Brink & Partridge, 1980; Partridge & Watt, 1991). Such features are often targeted during palaeontological excavations, as decalcification disentangles faunal remains from compact palaeocave breccia. At Drimolen, makondos are clearly associated with tree roots (Fig. 3C) and are typically filled with soil. Alternatively at Sterkfontein, makondos have decalcified ancient fossil-bearing palaeocave sediments (StW 53: Partridge & Watt, 1991). In these cases, it is important to clarify whether makondos are filled with younger, reworked paleocave fill, or represent more recent dissolution phases that have decalcified ancient, in situ palaeocave fill.

There is a suite of complex processes that can influence the formation of palaeocave stratigraphies. Allogenic sediments, bones and archaeological material are generally deposited fluvially and/or by colluvium, developing a range of facies related to changes in energy-related flows. Within the cave system, sediments can undergo multiple post-depositional processes including geogenic; both chemical (i.e., cementation-dissolution, phosphatization related to guano deposits), and mechanical (erosion, re-mobilisation and reworking); or biogenic (biological activity, reworked skeletal components) (Goldberg, 2000; Goldberg, 2001; Goldberg & Bar-Yosef, 2002; Karkanas & Goldberg, 2010; Karkanas & Goldberg, 2013; Karkanas et al., 1999; Karkanas et al., 2000; Macphail, Goldberg & Linderholm, 2000; Stoops, Marcelino & Mees, 2018). High fracture density resulting in increased water percolation and/or fluid introduction in caves, can result in high-energy channelised flows and localised chemical modification. Alternatively, ceiling collapse can result in the development of large talus cones, that have little evidence of fluvial transport during deposition, but are frequently associated with colluvia of fine clastic sediment and fossil remains. Contemporaneous, though lithologically different facies can grade into each other or occur in separate locations in the cavern, producing distinct lithological units that are in fact synchronous (Latham et al., 1999; Forbes & Bestland, 2007). On the other hand, chronologically separate facies may form under similar depositional environments and consequently be lithologically similar. Newer phases of karstification—polycyclic or polygenetic (*sensu* Ford & Williams, 1989: p.506)—can also affect older sediments of very different age within a stratigraphic sequence (refer to Fig. 3C), occasionally causing subsidence and collapse into younger chambers (Stratford, Grab & Pickering, 2014). These karstic processes and complex depositional histories are further complicated by extensive speleothem mining throughout the late nineteenth and early twentieth century, that has obscured or removed stratigraphic context (i.e., ex-situ mining deposits: Sterkfontein (Brain, 1981), Swartkrans (Brain, 1981) and Gondolin (Menter et al., 1999)). Consequently, many fossils recovered from the South African palaeokarst were *ex-situ* and their stratigraphic location has been reconstructed, or assumed (Dart, 1925; Broom, 1938; Brain, 1981; Herries & Adams, 2013; Herries 2003). As such, a suitable assessment of karstification, depositional and post-depositional factors are necessary when selecting strategies for assessing palaeocave sites.

### Methodological approaches to assessing palaeocaves

Traditionally, the stratigraphy of the classic CoH sites (Swartkrans, Sterkfontein, Kromdraai) has been defined by a lithostratigraphic approach (e.g., grainsize, colour and matrix, <15% clast vs. clast supported lithotypes) with different lithologic units defined as ‘Members’ (Butzer, 1976; Brain, 1976; Partridge, 1978; Partridge, 1979; Partridge, 2000; Bruxelles et al., 2016; Pickering & Kramers, 2010). ‘Members’, traditionally employed in partitioning the South African palaeocave deposits are by definition not necessarily isochronous North American Commission on Stratigraphic Nomenclature (2005) as is typical in multiple archaeological and palaeoanthropological settings. The discontinuous nature of the stratigraphy at many sites, as well as the inability to date members when they were defined, have also caused several issues when assessing the stratigraphy of the South African palaeocaves. Consequently, some defined members (e.g., Member 2, 3 and 4 at the Makapansgat Limeworks) have been contemporaneously deposited, and therefore represent lateral facies changes, where different members represent distinct, though often interconnected, depositional processes occurring simultaneously in different parts of the cave (Latham et al., 1999; Latham, Herries & Kuykendall, 2003; Herries & Adams, 2013). Inconsistencies also arise when attempting to correct previously numbered members that are later deemed inaccurate. Partridge (2000) redefined his original 1979 stratigraphy at the Makpansgat Limeworks by reclassifying Member 4a as Member 4 and Member 4b as the Central Debris Pile, mixing numbered members with named units. Latham et al. (2007) created a Member X between Members 1 and 2 at the Makapansgat Limeworks. At Kromdraai B Bruxelles et al. (2016) modified the original member system of Partridge (1982) by renumbering Members 3–5 into Member 4.1–4.3. Yet more recently, Ngoloyi et al. (2020) converted this numbering system to a lettered system (e.g., Unit P). Such adjustments in nomenclature can cause inconsistencies within the literature and can cause serious issues in associating individual fossil specimens excavated in the past, to the modern stratigraphic units. Braga et al. (2016), for example, proposes that it is now impossible to associate material excavated in the 1950’s at Swartkrans to any specific unit. To add to this, the term ‘Member’ is defined as next in rank below a geological formation and thus, ‘unit’ or ‘facies’ is a more appropriate term in these contexts North American Commission on Stratigraphic Nomenclature (2005). As various contemporaneous facies likely grade into each other, designating facies boundaries is often difficult and when possible, determining geochronological histories (based on; Uranium-lead [U-Pb], electron spin resonance [ESR], palaeomagnetism and cosmogenic burial dating) is crucial. At multiple sites, fossil-bearing palaeocave fills can be subdivided into allostratigraphic units (North American Commission on Stratigraphic Nomenclature, 2005), whose boundaries are underlain and/or capped by U-Pb dated flowstones that grow in stratigraphic continuity on the underlying sediment. Consequently, the allostratigraphic boundaries of the resulting Flowstone Bounded Units (FBUs) are isochronous as well (Herries et al., 2006; Pickering et al., 2007; Pickering et al., 2019; Herries et al., 2020). Moreover, Pickering et al. (2019) have shown that similar aged flowstones formed in multiple caves throughout the region and can therefore be used like marker beds between caves. However, at some sites interbedded and capping flowstones do not occur, or have been removed by erosion and so this is not always possible (Herries et al., 2018).

While stratigraphy is a fundamental concept understood by archaeologists and palaeoanthropologists, understanding it dictates an in-depth understanding of the site’s formation processes, as the site is an active part of the landscape (Ward, Winter & Dotte-Sarout, 2016). This is vital to selecting ideal excavation methods and subsequent laboratory analysis, and arguably, the more complex the stratigraphy, the more this principle is applicable. In complex palaeocave sequences, it is first critical to assess the geometry and stratigraphic order of the units in order to assess their stratigraphic relationships; second, to identify any post-depositional modification or disturbance—natural or anthropogenic—to develop a sound record of the site (Bruxelles et al., 2014; Dirks et al., 2010; Dirks et al., 2017; Stratford et al., 2012; Stratford, Grab & Pickering, 2014). Once this is achieved, geochronometric age determination (but also evolutionary inference) is required to reinforce stratigraphic relationships and date archaeological and palaeoanthropological material (Herries et al., 2018; Herries et al., 2020; Pickering et al., 2011b; Pickering et al., 2019). This approach still focuses on defining stratigraphic units; mostly litho- and allostratigraphic, based on stratigraphic, sedimentological and micromorphological observations, though also emphasises the importance of lateral and vertical changes that can be underpinned by chronological methods. This has been recently successful at recording age ranges for multiple deposits in the CoH (i.e., Rising Star: Dirks et al., 2017 and Drimolen Main Quarry: Herries et al., 2020). This modern approach combining both litho—and chronostratigraphy is a deviation from the classic ‘Member System’ and is more suited to contextualising palaeocave infill.

The aim of this paper is to provide the first comprehensive stratigraphic and micromorphological interpretation to reconstruct the site formation history of palaeocave deposits, as well as interred fossil material at Drimolen Makondo (DMK). The discovery of DMK demonstrates the potential for locating fossil sites that have not undergone extensive mining in the CoH. To date, only preliminary stratigraphic and micromorphological data is currently published for DMK. Due to the well exposed sections, minor mining damage and its spatial association with Drimolen Main Quarry (DMQ: Fig. 3A), assessing depositional and post-depositional processes resulting in the formation of clastic fill at DMK is achievable. As interbedded flowstone deposits do not occur within clastic fill at Drimolen Makondo (Fig. 3A), determining stratigraphic relationships, including allostratigraphic and synchronous boundaries between clastic units is particularly important for the deposit. In expanding from preliminary work presented in Herries et al. (2018) at DMK and recent broader geological works in Murszewski et al. (2019) research here will also play a role in understanding the genesis and infill of the entire Drimolen palaeocave system. This is in addition to providing an opportunity to study a palaeocave site that encompasses the Pliocene to Pleistocene transition, which is a rare phenomenon in the CoH (Herries et al., 2019).

## Site Setting

The Drimolen fossil site (alt. Drimolen Palaeocave System) is located between ∼4–6 kilometers north of the Blaauwbank Valley sites (Fig. 1) and positioned at one of the highest dolomite exposures in the Gauteng Province (∼1545 masl). Recent geological assessments carried out surrounding Drimolen, indicate that the cave system is positioned within the most northerly extent of the Monte Christo Formation. The original site discovered in 1992, now referred to as Drimolen Main Quarry (DMQ), is a large palaeocave from which stone and bone tools, and hominin remains have since been recovered (Adams et al., 2016; Herries et al., 2020: Keyser et al., 2000; Stammers, Caruana & Herries, 2018).

**Figure 4:**
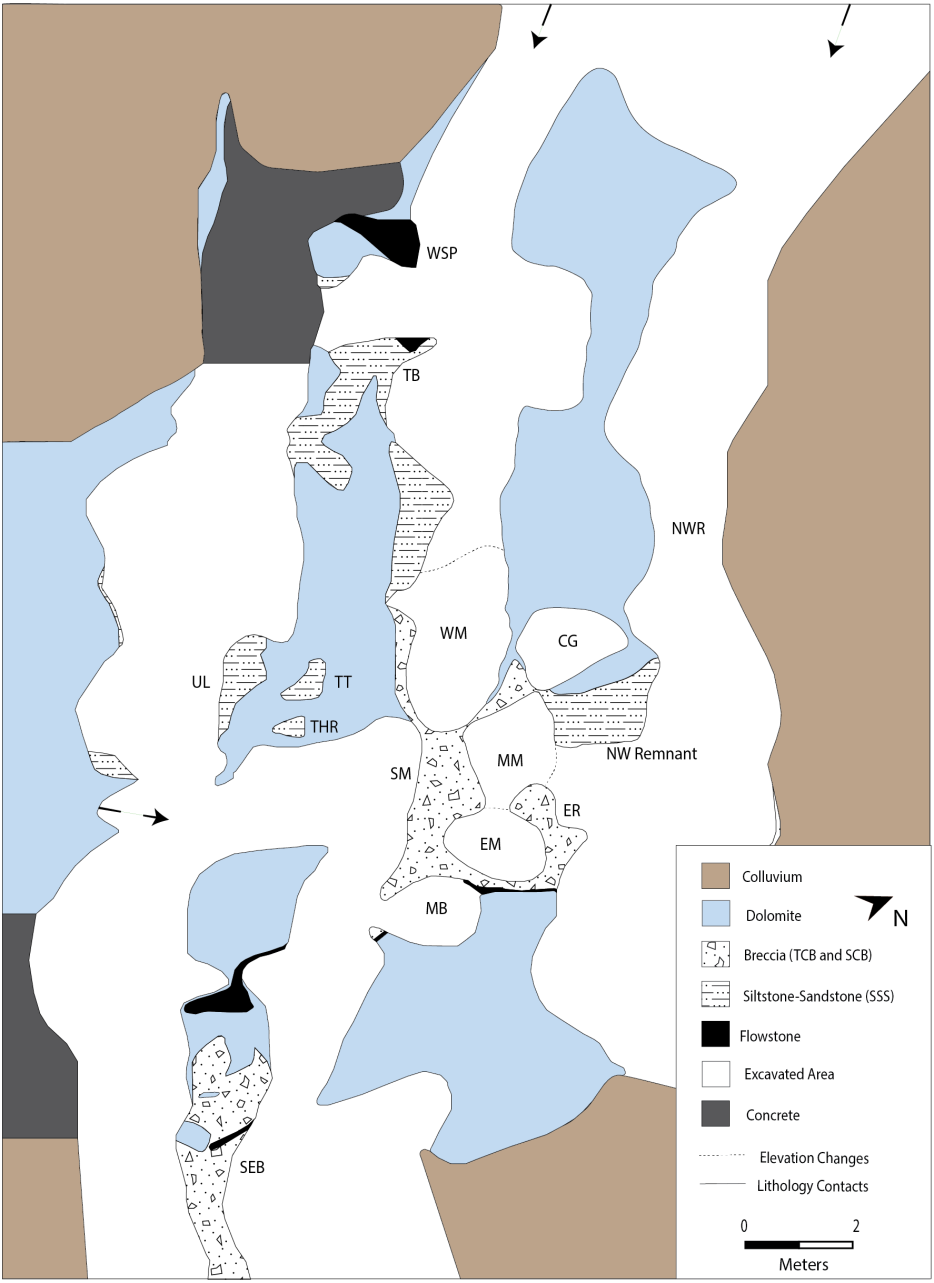
Simplified DMK site map showing locations of various makondos and pockets of sediment/soil fill respectively along palaeocave walls and within solution cavities. Dotted lines showing orientation of karstification channels. MM, Main Makondo; EM, Eastern Makondo; WM, Western Makondo; NM, Northern Makondo; SM, Southern Makondo; ER, Eastern Remnant; NWR, North West Rift; NW Remnant, North West Remnant; MB, Maddie’s Bath; CG, Chris’ Gym; TB, Traynorberg; TT, The Tongue; THR, The Hanging Remnant; UL, Uluru; SEB, South East Breccia; WSP, Western speleothem. Note: Unit presented is the primary lithology at the surface.

Exploration uphill of DMQ, identified a possible extension of the palaeocave system to the west within a small makondo (Brink & Partridge, 1980). Subsequent excavations since 2014 have uncovered a series of sediment and soil-filled makondos with a rich palaeontological record (Rovinsky et al., 2015) (Figs. 3B and 4), now referred to as Drimolen Makondo (DMK; Rovinsky et al., 2015; Herries et al., 2018). A series of more recent actively infilling cave systems have also been identified, such as: Porcupine Cave (Fig. 3A), which has formed beneath DMK; and Warthog Cave, which has formed between calcified palaeocave sediments and dolomite on the western edge of DMQ. Based on cosmogenic erosional rates from quartz samples collected on the hill to the west of the Drimolen palaeocave complex, it is estimated that ∼26 m of deposits and overlying dolomite have been eroded over the last 2.61 Ma (Herries et al., 2020).

At DMK, small sections along the North West Rift (Fig. 4) were exposed during discontinued lime mining tests, although this was not extensive enough to remove stratigraphic associations or impact fossil deposits. Makondo formation has also removed a large portion of palaeocave fill, however this process has also exposed deep stratigraphic sections, significantly aiding contextual studies. In some cases, this has caused decalcification of palaeocave infill without major reworking, shown through articulated fossils occurring *in situ* across both decalcified and calcified deposits (Herries et al., 2018; Rovinsky et al., 2015). In other cases, decalcified material within makondos have likely been reworked via collapse into lower caverns. An example of this is Porcupine Cave; a more recent cavern that is connected to the base of breccia deposits at Eastern Makondo at DMK (Figs. 3A and 4). These makondos were subsequently infilled with colluvium, which covered over 90% of the deposit prior to excavation in 2014.

The basal flowstone in both DMK and DMQ formed at ∼2.7 Ma (Pickering et al., 2019; Herries et al., 2020). However, sediment infilled DMK at ∼2.61 Ma soon after the DMK formation of the basal flowstone, based on US-ESR combined with U-Pb and palaeomagnetism (Herries et al., 2018). This is unlike DMQ, where sediments infilled the cave significantly after the formation of the basal flowstone (∼600 ka) (Herries et al., 2020). Chronological separation is also shown by the different fauna from the two deposits with Stage 1 *Metridiochoerus andrewsi*, *Dinofelis barlowi* and *Parapapio* fossils from DMK and both *D. barlowi* and *Dinofelis aff. piveteaui* as well as *Equus* and *Papio robinsoni* fossils from DMQ (Adams et al., 2016; Herries et al., 2020).

## Materials and Methods

All fieldwork was conducted under the South African Heritage Resources Agency (SAHRA) Permit ID 2035. Macroscopic characteristics of sediments and stratigraphic relationships between facies were observed and described on natural profiles (i.e., dissolution surfaces within and around the makondos), as well as on recent excavation surfaces of decalcified material and exposed mining surfaces. Areas of excavation were constrained and named according to solution cavities or shapes of adjacent breccia walls (Fig. 4). Fieldwork descriptions and stratigraphic analysis were carried out following the methodology indicated by Catt (1990). Basic grainsize estimates were performed in the field, identifying general vertical and lateral trends throughout the site. Understanding the architecture of sedimentary bodies and reconstructing infill history were the primary focus during onsite macroscopic analysis, with emphasis given to texture, boundaries, sedimentary structures within the strata. Weight was given to trends and variations in stratigraphic sequences to define litho- and allostratigraphic units. Vertical and lateral changes are prominent throughout the DMK sequence, and inform depositional and post-depositional processes at the site. Micromorphological analysis allows for accurate and in-depth determination of sedimentary dynamics (inc. microscopic sorting, grading, lamination, reworking) (Karkanas & Goldberg, 2013). Therefore, a series of micromorphological samples were extracted for subsequent laboratory analysis. Stratigraphic boundaries on various profiles were subdivided. We define these by: (1) major facies; an entity distinguished by depositional process; and (2) sub-facies, that document changes within facies. Micromorphological samples were extracted to best define micromorphological components within each of the major facies and speleothem formations at DMK. Spatial information was recorded for each sample, while also photographed and documented *in situ*. Emphasis was given to facies and sub-facies that are exposed in walls among the central solution cavities, including, Main Makondo, Eastern Makondo, Eastern Remnant and North West Remnant (Fig. 4).

All field samples were extracted from exposed profiles by hammer and chisel or angle grinder. Laboratory thin section preparation followed FitzPatrick (1984), where the remainder of each sample was kept for future geochemical or sedimentological analyses. Thin section observations and microphotography were carried out using a Zeiss polarising petrographic microscope. Descriptions follow standardised terminology (Stoops, 2003), where identification and interpretation of components and pedofeatures are based on the micromorphology literature available, both in a general sense (Bullock & Thompson, 1985; Courty, Goldberg & Macphail, 1989; Goldberg & Berna, 2010; Stoops, Marcelino & Mees, 2018) and from cave sites (Boschian, 1997; Karkanas et al., 2000; Karkanas & Goldberg, 2010; Karkanas & Goldberg, 2013). More extensive literature sources are cited in text. Aspects that were of primary focus for this research include those characteristics that can be used in reconstructing sedimentary processes and identification of post-depositional processes (i.e., optical properties of minerals, sorting, fining or coarsening upwards sequences, microstructure and pedofeatures, organic components, bioturbation, reworking and cementation-dissolution of clastic fill).

## Results

Recent work at DMK has concentrated on removing recent colluvium to assess *in situ* breccia and its decalcification products. Excavations have also exposed dolomite bedrock in the far western area of the site and on ridges separating multiple dissolution channels. These channels were originated by ceiling collapse/dissolution over former cave passages that are aligned predominantly WNW (Fig. 4). This layout shows the cave consisted of a network of intercommunicating passages (Davis & McMillan, 2017), similar to the adjacent Porcupine Cave. CaCO_3_-cemented clastic units adhere to the walls of dolomite in multiple locations and are divided into three main macro-scale depositional facies (*sensu* North American Commission on Stratigraphic Nomenclature, 2005: art. 22, p. 1567), according to previously observed field evidence (Pickering et al., 2007; Herries et al., 2020) (Table 1); F1: talus cone breccia (TCB), F2: reworked talus cone breccia (SBC) and F3: fluvial sandstone-siltstone deposits (SSS) (Fig. 4). Sub-facies within SBC were best defined on changes in clast size.

**Figure 5:**
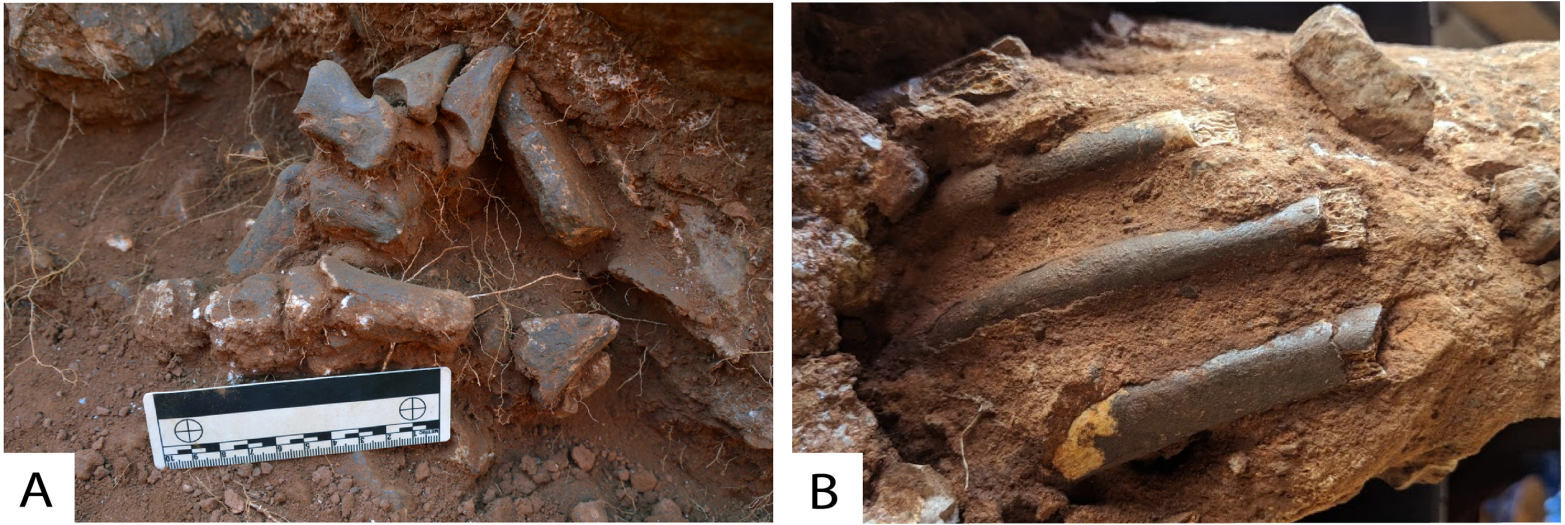
Partially articulated fossil remains within Drimolen Makondo. (A) Partially articulated bovid foot exposed during excavations within Eastern Makondo. (B) Partially articulated ribcage excavated within Eastern Makondo. Photograph *ex situ*.

More recent makondo features have eroded a significant portion of clastic material from each of these depositional facies at DMK. Prominent fining and coarsening upwards trends within SBC, are well-preserved within the central extents of the site (i.e., Main Makondo, Eastern Makondo, Eastern Remnant: Fig. 4). Decalcified palaeocave sediments with partially articulated macrofossil remains are also well preserved within central makondo features (Fig. 5). Partially articulated fossil remains can be traced from within the breccia to the decalcified material, indicating that the latter is largely *in situ* (Herries et al., 2018). A deep artificial trench previously filled with speleothem and wad occurs on the northern side of DMK (Fig. 4). This is the only section of the site that has been minimally mined and does not influence interpretations presented here. Field observations and stratigraphic work focused along these vertical profiles at DMK, and are supplemented with micromorphological observations for each of the major depositional facies (Table 2). These are described hereafter.

**Table 1:** Major depositional facies (and sub-facies) of DMK clastic fill.

Clastic Facies	*Sub-facies*	Description	Sample ID
1 TCB		Facies is composed of unsorted dolomite, chert and to a lesser extent shale (from fine pebble to coarse boulder size [up to 60cm]), within a reddish- brown, silty loam matrix. Facies is typically clast supported, though subordinately shifts to matrix-supported, with dolomite clasts typically sub-angular to sub-rounded and clast and shale clasts generally platy or angular to sub-angular. Facies strongly cemented by calcite (CaCO_3_).	DMKMM07; DMKMM09
2 SBC		These deposits are characterised by wider grain size ranges from large chert or dolomite clasts (typically less than 30cm), where the finer fraction is organised into laminae constituted with clay- to fine pebbles. This finer fraction preserves bedding features, fining- and coarsening-upwards lenses, where sub-facies (a –f: below) highlight changes in clast size and fabric. Grain shape varies from angular to sub-rounded. Facies cemented by calcite (CaCO_3_).	
	*a*	Coarse fraction composed of chert, and to a lesser extent dolomite, ranging from large cobbles and pebbles within a reddish- brown, silty loam matrix, composed of clays- to granules. Unit poorly sorted, with wide spaces amongst coarse clasts and macro-fossils (i.e., typically matrix-supported). High density of bone preserved within unit.	
	*b*	Typically, clast-supported. Coarse fraction composed of chert and dolomite, ranging from boulder (up to 40cm) to pebbles, poorly sorted within a reddish- brown, silty loam matrix, composed of clays- to granules.	DMKMM08
	*c*	Matrix-supported breccia. Coarse fraction mainly composed of chert and dolomite pebbles and to a lesser extent, cobbles (up to 10cm), moderately sorted within a silty loam matrix consisting of clays- to granules. Laminations of this finer material, intercalated by coarser lenses, frequently observed.	DMKMM06
	*d*	Clast-supported. Coarse fraction composed of chert and dolomite, ranging from large cobbles and pebbles within a reddish- brown, silty loam matrix, composed of clays- to granules. Unit poorly sorted, with coarse clasts and fossils more frequently compacted, with wider spaces amongst clasts less frequent.	DMKMM03; DMKMM05
	*e*	Matrix-supported. Well sorted, laminated clasts within the silt- and sand size fractions with fine pebble inclusions. Larger pebbles and cobbles identified and are also characteristic of this unit.	DMKMM04
	*f*	Matrix-supported, though subordinately shifts to matrix-supported. Coarse fraction composed of chert and dolomite, ranging from boulders to pebbles (up to 40cm) within a reddish- brown, silty loam matrix, composed of clays- to granules. Unit very poorly sorted, with coarse clasts and fossils dispersed, with wider spaces between clasts frequent.	DMKMM10
3 SSS		Facies are relatively homogenous throughout DMK, with minor changes related to grain size fluctuations. The average grainsize is smaller than that of facies 1 or 2, and consists of well sorted, laminated clasts within the silt- and sand size fractions with very few fine pebble inclusions. Intercalations of fine speleothem crusts frequently observed. Sediments are organised into laminae and layers less than 5cm thick, in sub-horizontal, fining upwards sequences typically terminated by thin lenses of clay, or thin crusts of speleothem. Micro-mammalian bone very common.	DMKMM14; DMKMM15

**Table 2:** Micromorphological aspects of each of the major facies at DMK.

	Overview	Microstructure and porosity	Groundmass		Organic components	Pedofeatures
			Basic mineral components	Fine fraction (*inc. micromass*)		
Wad	Crumb microstructure, with Fe- and Mn- oxide hypocoatings along carbonate crystals. Clastic fraction typically clustered.	Well separated crumb microstructure.	Poorly sorted, rounded to sub-rounded monocrystalline and polycrystalline quartz, chert and micaceous fragments (<500 µm).	Fine monocrystalline quartz and clays stained by amorphous Fe- oxides.		Interlocking anhedral to subhedral calcite. Mn-oxide coatings and hypocoatings along relict carbonate crystals. Fe- and Mn- coatings and hypocoatings on clastic material.
Basal Flowstone (BFS)	Radial aragonite growths within a mosaic of subhedral and anhedral calcite.		Little to no detrital material. Very fine, opaque angular Fe- and Mn oxides infrequent.			Alternating zones of radial aragonite within a mosaic of anhedral and subhedral calcite. Relict columnar and radial aragonite crystals preserved in calcite fabrics.
TCB	Granular microstructure to crumb microstructure. Coarse fraction unsorted within a reddish-brown sandy to silty loam matrix; very strong CaCO3 cementation. Various alteration of clastic material.	Granular microstructure most common, however well-developed crumb microstructure also observed.	Unsorted, angular to sub-angular monocrystalline and polycrystalline quartz, chert, dolomite and subordinate micaceous clasts. Aggregates typically composed of fine quartz grains and heavily stained by Fe- and Mn- oxides.	Reddish-to brown sandy to silty loam matrix. Includes fine sand-sized to silt clasts and aggregates of clay and amorphous Fe- and Mn- oxides. B-fabric masked by amorphous iron-oxides.	Micro-mammalian bone and tooth fragments.	Strongly cemented by a mosaic of anhedral to subhedral calcite. Relict crystal boundaries frequently observed. Push-apart features somewhat frequent, where clastic fraction has been pushed in the direction of calcite growth. Cyclical clay coatings (alt. clay cutans).
SBC	Granular microstructure. Coarse fraction poorly sorted within a reddish-to-brown sandy to silty loam; CaCO_3_ cementation. Various alteration of clastic material.	Granular microstructure. Complex packing and channel voids.	Very poorly sorted, angular to sub-angular monocrystalline and polycrystalline quartz, chert, dolomite and subordinate micaceous clasts. Aggregates typically composed of fine quartz grains and heavily stained by Fe- and Mn- oxides.	Reddish-to brown sandy to silty loam matrix. Includes fine sand-sized to silt clasts and aggregates of clay and amorphous Fe- and Mn- oxides. Poorly developed stipple-speckled b-fabric partly preserved.	Micro-mammalian bone and tooth fragments. Macro-mammalian bone fragments. Coprolites (subcircular with amorphous composition) Woody tissue fragments. Pollen (*Podocarpus*).	Cemented by a mosaic of anhedral to subhedral calcite. Fe- and Mn- oxide coatings and hypocoatings common on grain and clast boundaries. Rounded to sub-rounded Fe oxide nodules.
SSS	Granular microstructure. Reddish-to-brown sand to silty loam; CaCO_3_ cementation. Parallel layering of thin layers; frequent fining-upwards sequences terminated by thin clay crusts or fine calcite lenses.	Granular microstructure. Channel voids dominant, complex packing voids and chambers also observed.	Moderately to well sorted, angular to sub-rounded monocrystalline and polycrystalline quartz. Chert, dolomite fragments are observed to a lesser extent. Sub-rounded aggregates, typically composed of very fine silts and clays.	Includes fine sand-sized to silt clasts and aggregates of clay and amorphous Fe- and Mn- oxides. Poorly developed stipple-speckled b-fabric partly preserved.	Micro-mammalian bone and tooth fragments very common. Woody tissue fragments.	Fe- and Mn- oxide coatings and hypocoatings common on grain and clast boundaries. Fine sparitic coatings on bone surfaces. Mn-staining prevalent on woody tissue and bone fragments. Fine, rounded to sub-rounded Fe oxide nodules. Very few, rounded calcitic nodules.
Western speleothem	High degree of variability in precipitation cycles, including interwoven centripetal calcite, columnar calcite and radial aragonite growths. Variable thickness (5 –1cm) on the vertical surface.		Very little detrital material. Few aggregates composed of fine monocrystalline quartz, Fe-oxides and clays.	Fine silts and clays at the interface between microbands of anhedral calcite.		Initial precipitation phases characterised by microbands of fine calcite crystals and coarse equant mosaic calcite. Later precipitation highly variable and include interwoven centripetal calcite, columnar calcite and radial aragonite growths.

### Wad

**Figure 6:**
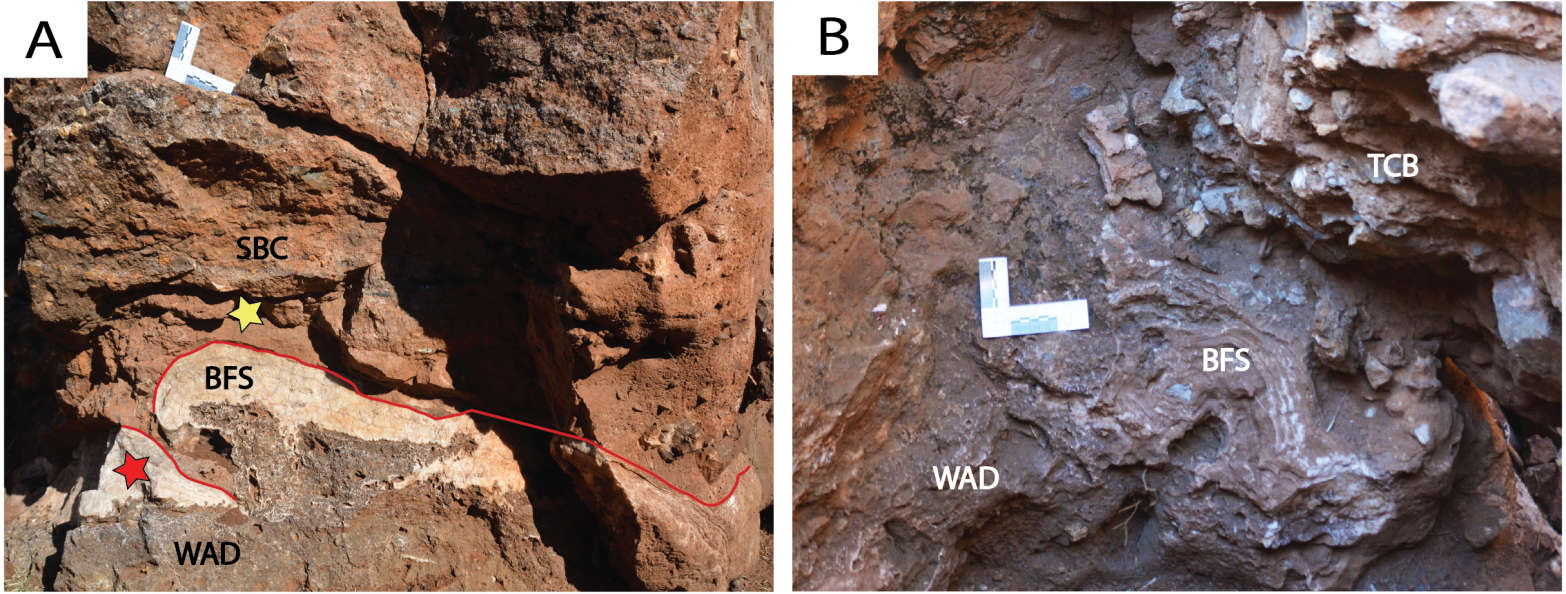
Various aspects of the basal flowstone at DMK. (A) Basal flowstone (BFS) with underlying wad (WAD) in the far north-western exposures of the North West Remnant. Reworked breccia deposits (SBC) overlie basal flowstone. Red star—location of U-Pb sample. Yellow star—location of palaeomagnetism sample. (B) Basal flowstone (BFS) with underlying wad (WAD) at the base of eastern profile in Main Makondo. Overlying talus cone breccia (TCB) deposits shown.

**Figure 7:**
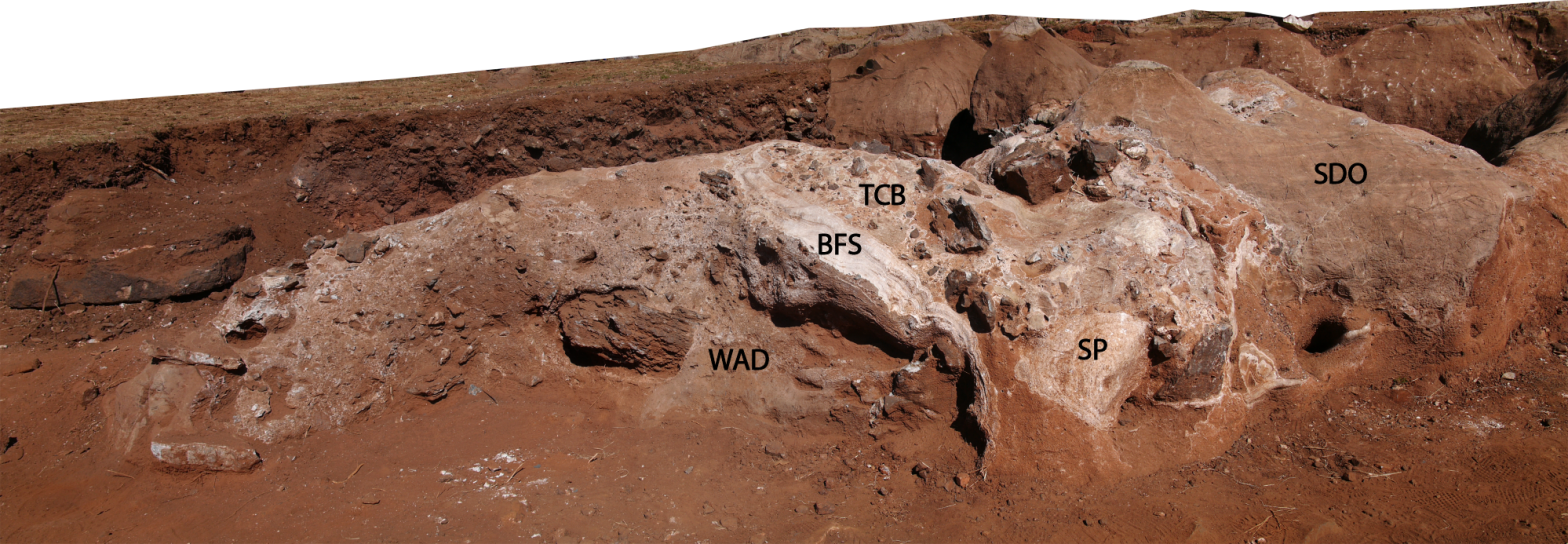
*South-East Breccia*. Calcite precipitation and basal flowstone preservation (BFS) overlying wad (WAD) and dolomite (SDO). Large angular chert boulders, along with dolomite and chert clasts of various sizes within a fine, reddish-brown matrix (TCB). Remnants of a speleothem boss also preserved (SP). Area isolated from primary talus located in Main Makondo.

**Figure 8:**
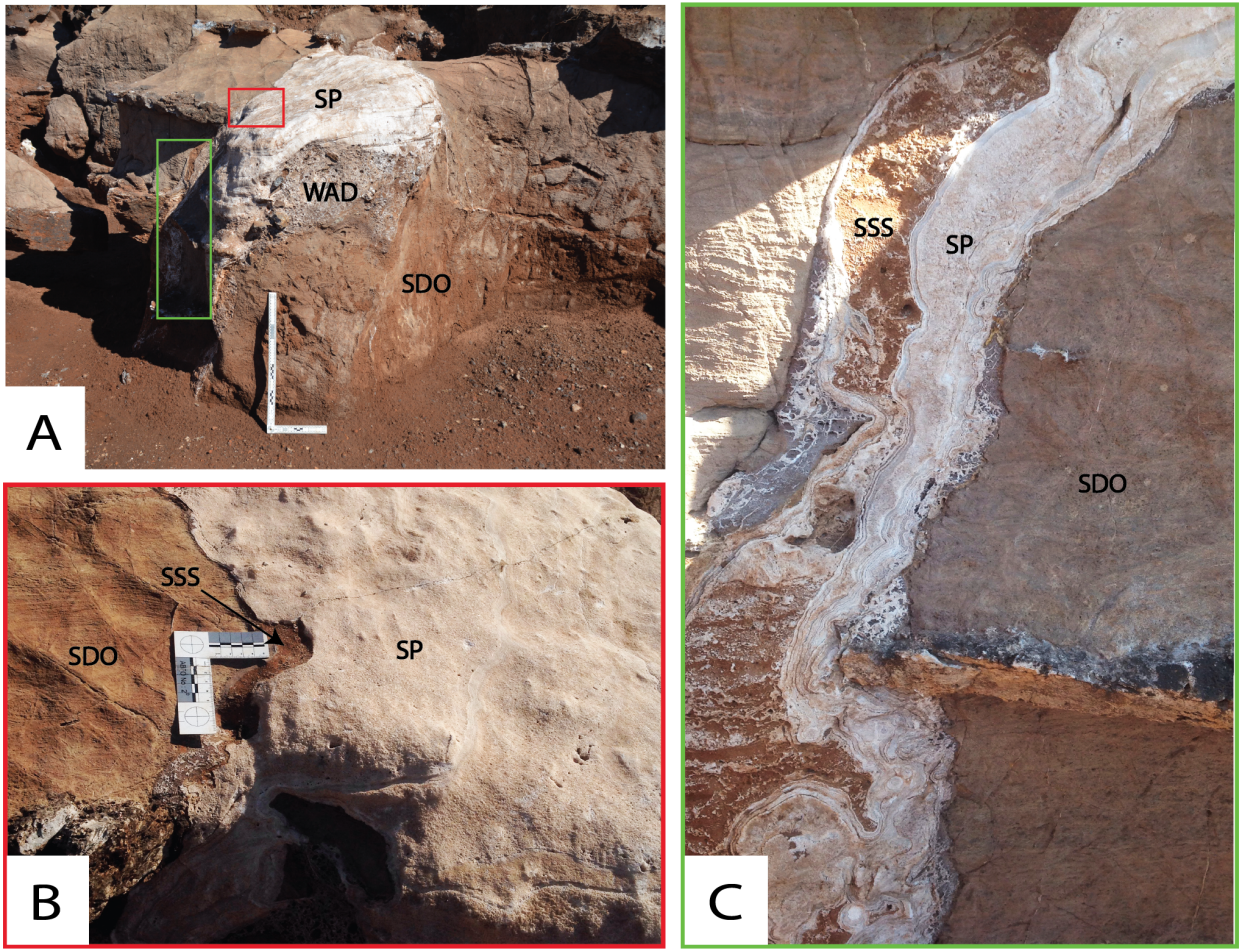
Various aspects of the Western Speleothem. (A) Image of Western Speleothem facing west (ref. to Fig. 4 for spatial association to the rest of the site), indicating dolomite (SDO), basal wad (WAD) and speleothem (SP). (B) Contact between dolomite (SDO), siltstone-sandstone (SSS) and overlying speleothem (SP). (C) Contact between siltstone-sandstone (SSS), overlying speleothem (SP) and dolomite (SDO) at the vertical continuation of the speleothem (facing north-west).

**Figure 9:**
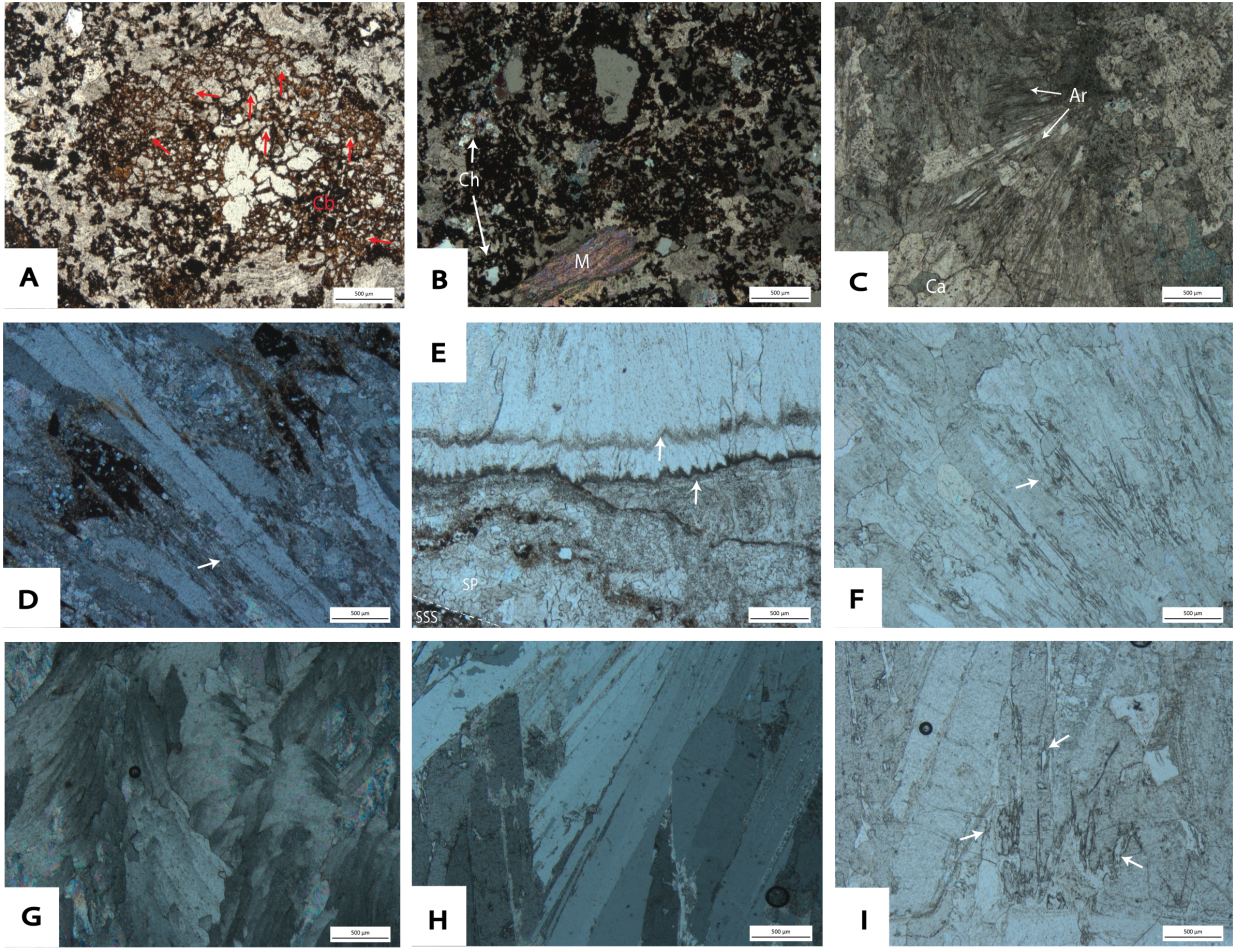
Photomicrographs taken from thin sections at DMK. (A) Manganese ‘wad’ sample: DMKMM16 (PPL), showing loose Mn- aggregates within interlocking calcite infills. Mn-oxide hypocoatings on dolomitic crystals (cb) present with accumulation of Fe- and Mn- oxides along relict carbonate crystals. (B) DMKMM16 (XPL): clastic material present in clusters of Mn- and Fe- oxides within basal wad deposits. Ch, chert clasts. M, micaceous fragment with high order birefringence colours. (C) Basal flowstone: DMKMM11 (XPL), showing fan-like aragonite relicts (Ar) within mosaic anhedral calcite (Ca) crystals. Triple points frequent. (D) Zones of relict-elongated aragonite crystals within basal flowstone sample DMKMM21 (XPL) from the South East Breccia. (E) DMKMM17U (PPL), of the contact zone between SSS units and the western talus at the far western portion of the site. Minimal clastic material observed at the contact, which consists of microlayers of calcite with scalenohedral terminations (arrow). (F) DMKMM18 (PPL) showing acicular voids; pseudomorphs of pre-existing fan-like aragonite crystals. (G) Pseudomorphs of interwoven, centripetal aragonite growths, built of calcite within sample DMKMM17U (XPL). (H) Large elongated bands, showing relict aragonite fabrics within DMKMM17U (XPL). Acicular voids present along scalenohedral terminations. (I) DMKMM17U (PPL), showing fan-like acicular voids from pre-existing aragonite growths within speleothem.

Wad (alt. wadstone) is a term used to define manganese hydroxide deposits (Wenk & Bulakh, 2004; p. 423), typically used in South Africa to describe dark brown insoluble residuum remaining after dolomite dissolution (Martini & Kavalieris, 1976). Extensive wad deposits are concentrated around the NW Remnant (Fig. 6), South East Breccia (Fig. 7) and the Western Speleothem (Fig. 8) at DMK. Wad deposits are in contact with dolomite, where they likely formed, and are overlain by speleothem and are characterised at DMK by a network of calcite veins filling the voids among aggregates, which are visible at macroscopic scale (Fig. 8). At the micro-scale, wad deposits have a dense crumb microstructure, composed of Fe- and Mn-aggregates and fragments of heavily degraded dolomite clasts. Abundant Fe- and Mn-oxides released by dissolution concentrate along carbonate crystals and frequently form hypocoatings on relict dolomite crystals (Fig. 9A). Zones of clustered, sub-rounded chert clasts, well preserved micaceous fragments and clay are also present (Fig. 9B). Though a large portion of this material derives from *in situ* dissolution of dolomite, some may have been introduced by percolation along rock joints.

### Speleothem

Multiple spatially distinct speleothem deposits are observed throughout DMK profiles. The first is the basal flowstone, which partially overlays residual wad along the base of the North West Remnant. The continuation of this basal flowstone can also be seen at the base of Main Makondo, at the base of the eastern profile (Fig. 6). The contact between the basal flowstone and overlying facies is sharp. Unconnected speleothem crusts are situated between wad or dolomite and clastic cave infill and likely formed synchronously with the basal flowstone. These are also observed at Eastern Makondo and Maddie’s Bath. Speleothem is also documented between wad and clastic palaeocave fill at the South East Breccia (Fig. 7), where the contact with the overlying clastic facies is also sharp and without evidence of backward dissolution. At microscopic scale, the basal flowstone commences with isolated pockets of fan-like, acicular, aragonite crystals that are partially preserved within a recrystallised mosaic of equant subhedral to anhedral calcite crystals (Fig. 9C). Acicular, or elongated voids and impurities are also frequently observed within calcite crystals. Direction of growth of relict aragonite indicates that this unit formed upwards with no major erosional phases identified. The basal flowstone exposed at the South East Breccia is similar to the other outcrops of basal flowstone, however it also preserves relict columnar aragonite fabrics of calcite crystals (Fig. 9D).

Speleothem observed at the far north western portion of the site (Fig. 8) overlies SSS units, thus is likely the youngest unit of the DMK sequence. However, dating is still required to corroborate this. The thickness of the flowstone varies from ∼5 cm at the top of the karstic dissolution pinnacle to <1 cm down the vertical surface (Fig. 8). The contact between SSS and speleothem along the vertical surface is characterised by microlayers of anhedral sparitc calcite (Fig. 9E). Subsequent microlayers are constituted by coarse equant mosaic calcite with scalenohedral terminations, indicating direction of crystal growth (Fig. 9E). Minimal detrital material is observed at the contact with the previous layers, where clay accumulated along crystal boundaries. Calcite belonging to later precipitation phases is pseudomorphic after aragonite, including acicular (Fig. 9F) interwoven centripetal growths (Fig. 9G), elongate columnar crystals (Fig. 9H), and fine, fan-like radial crystals and voids, typically observed along larger columnar crystal boundaries (Figs. 9H and 9I).

### Talus cone breccia (TCB)

**Figure 10:**
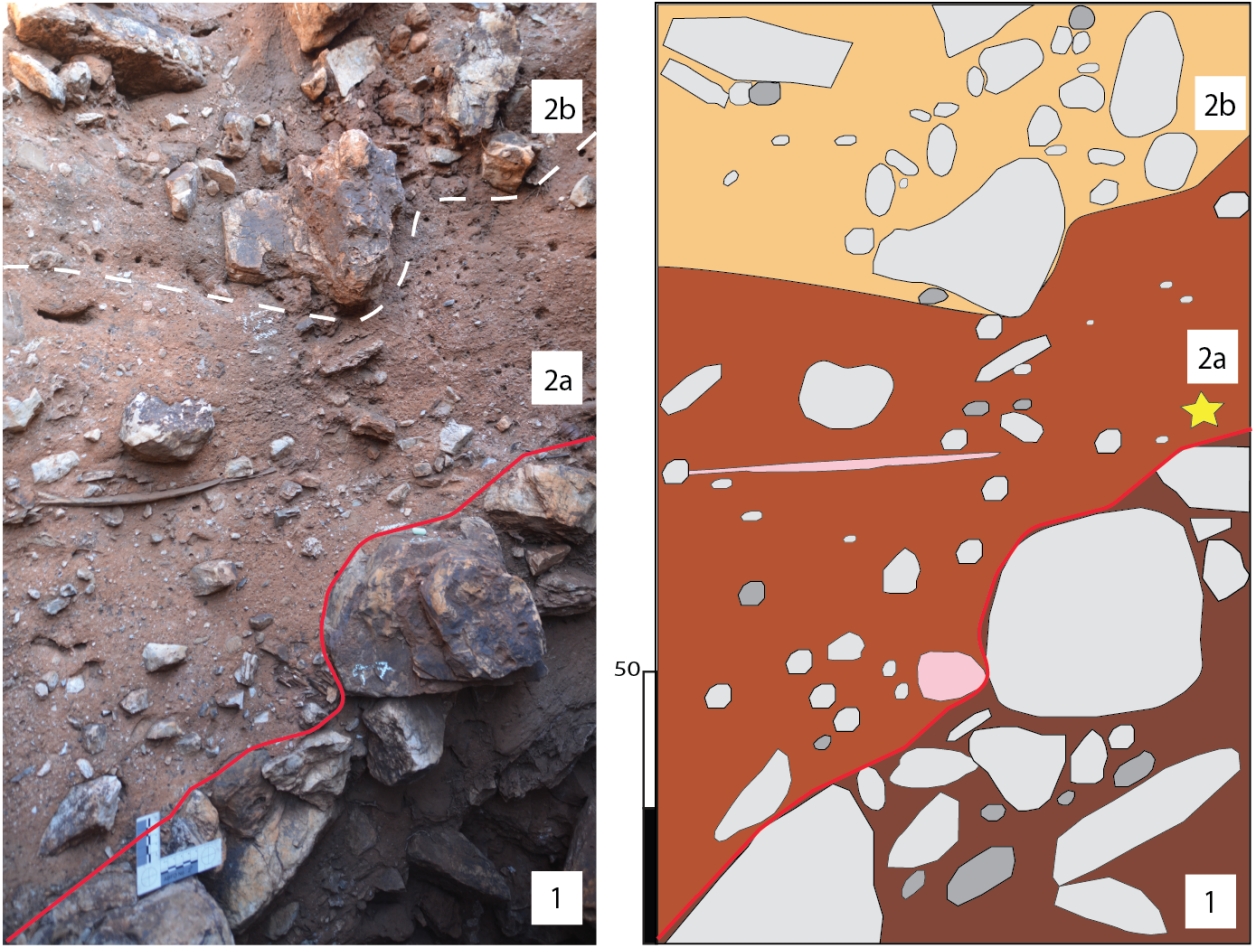
Main Makondo (western wall). Relationship between TCB (1) and SBC (2) shown, along with subfacies ([2] a and b). Top of TCB shows the NW dip angle of talus cone. Location of palaeomagnetic sample (yellow star) shown in figure.

**Figure 11:**
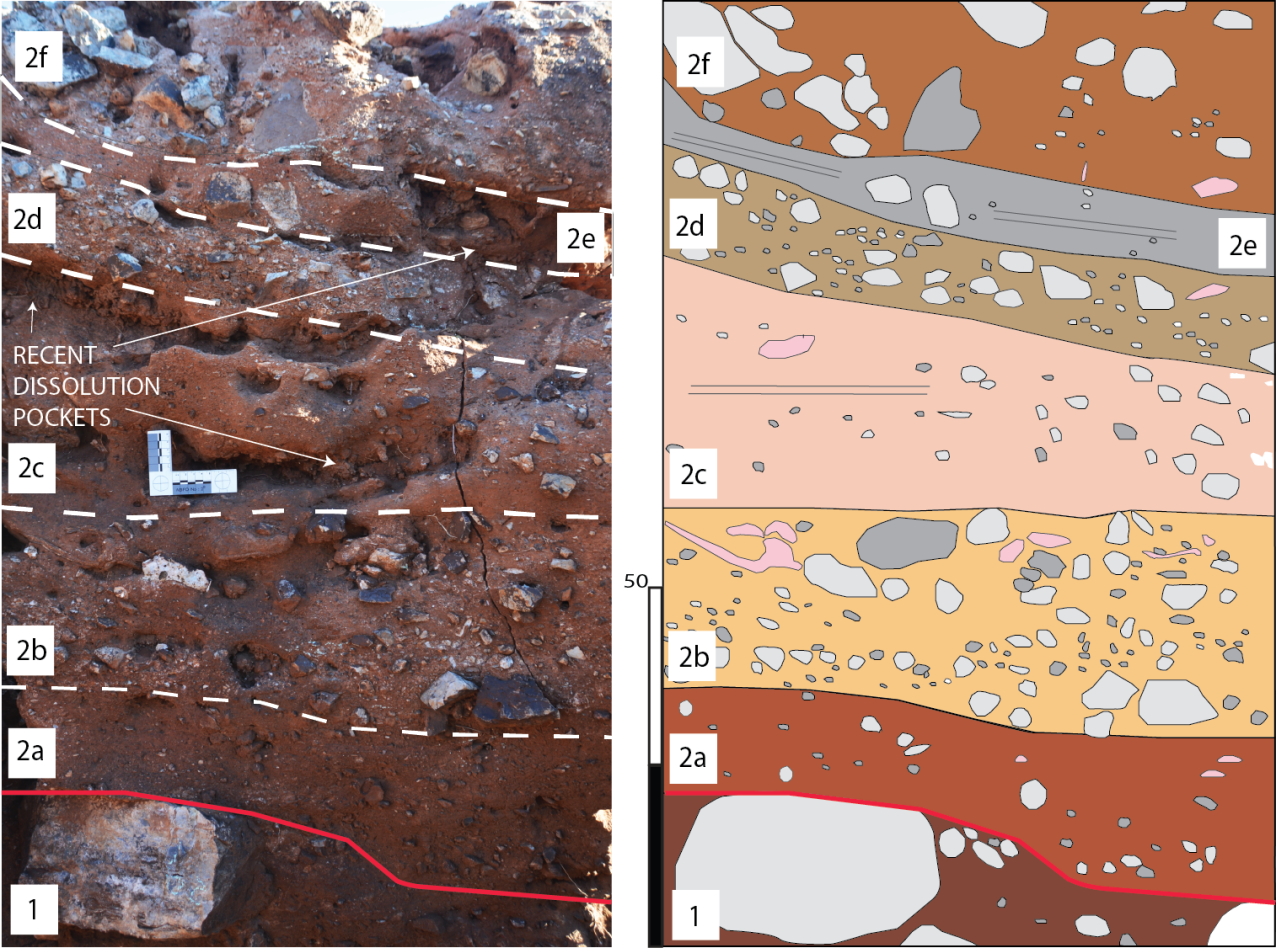
Eastern Makondo (eastern wall). Relationship between TCB (1) and SBC (2) facies shown, along with sub-facies within SBC distinguished in the profile.

The original talus cone at DMK is well-represented by the coarse blocks observed at the base of Main Makondo (Fig. 10). This material represents the only phase of talus sediments that have not been significantly reworked within the central exposures of DMK. The base of TCB is composed of clast-supported, planar chert boulders stratigraphically overlying the basal flowstone. These are orientated parallel and dip towards the south-west in Main Makondo (Fig. 10). Here, the upper contact is relatively sharp, indicating an erosional surface at the contact between TCB and SBC (2a) facies. Structure of TCB facies subordinately grades into matrix-supported towards the base of Eastern Makondo (Fig. 11).

TCB facies is typically composed of unsorted dolomite and chert clasts from pebble to large boulders (<30–40 cm) within a reddish-brown, silty loam matrix (Fig. 10). Clast shape is largely controlled by lithology, where dolomite clasts are typically sub-spherical and subangular/subrounded, and larger chert fragments are typically platy and angular to sub-angular. This is expected as dolomite breaks into sub-spherical shapes determined by layering and joint pattern, while large chert clasts originated from local planar chert layers interbedded between the dolomite. In addition, chert is typically more resistant to weathering by mechanical and chemical processes. This is also seen within decalcified breccia inside makondos. Here, dolomite clasts often degrade to localised grey sand patches while retaining the shape of the clasts, whereas intact chert clasts are still abundant.

**Figure 12:**
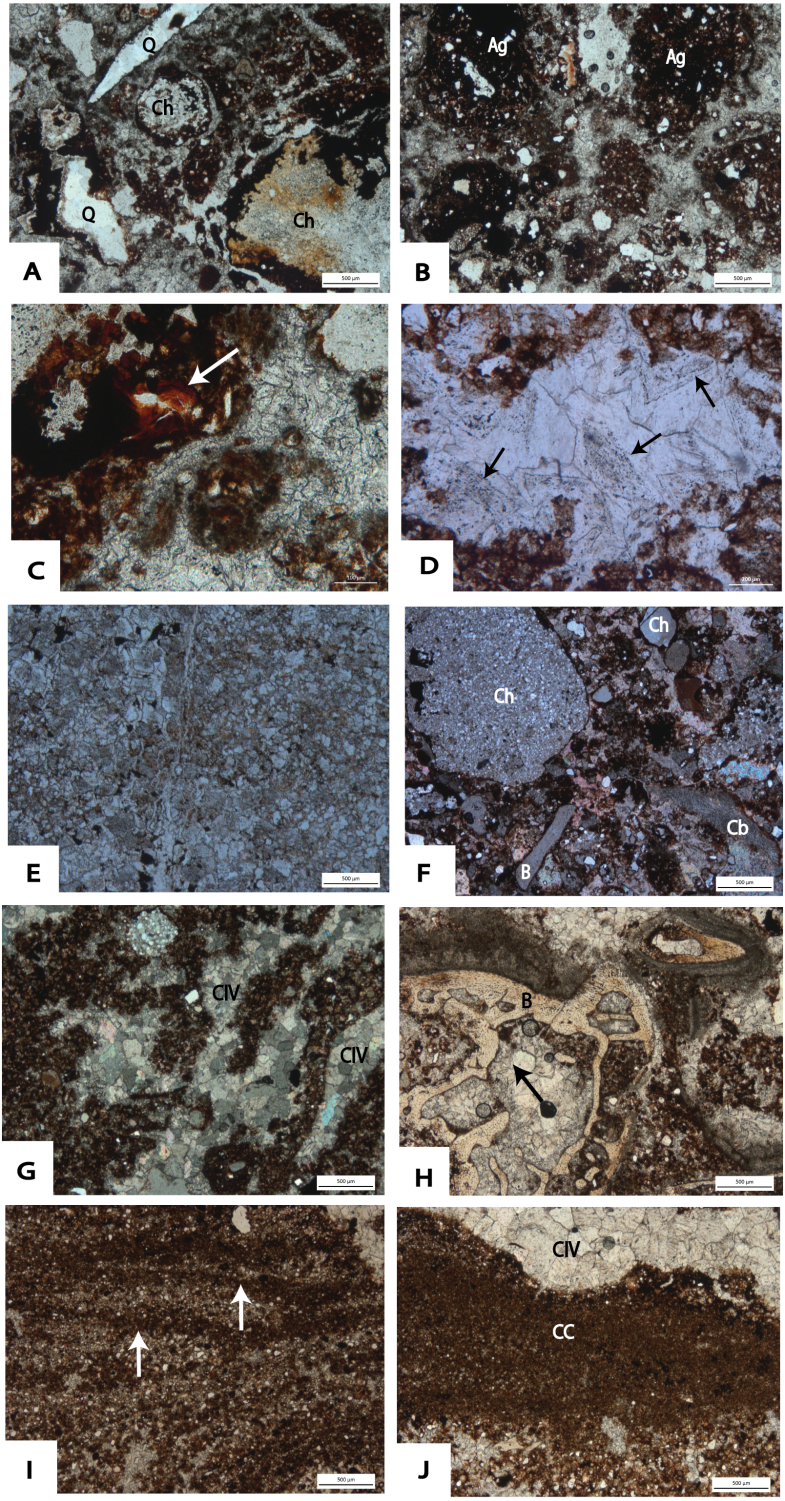
Photomicrographs taken from thin sections at DMK. (A) TCB: DMKMM09 (PPL), showing ‘loose’, crumb microstructure cemented with calcite. Composed of heavily eroded chert fragments, angular quartz fragments, and fine silts and clays in the fine fraction. (B) Crumb microstructure observed in DMKMM09 (PPL). Aggregates composed of monocrystalline quartz grains and dense Fe- and Mn- oxides, with fine voids observed. (C) DMKMM09 (PPL); clay cutans forming adjacent to ferruginous nodules and clays within a calcite matrix of TCB. (D) DMKMM07 (PPL) Relict euhedral calcite boundaries within void spaces at the top of TCB in Main Makondo. (E) DMKMM21 (PPL): Clay observed within the crystal boundaries within a heavily degraded dolomite boulder at the South East Breccia. (F) SCB: DMKMM08 (XPL), showing granular microstructure within a mosaic of calcite crystals showing pale high order interference colours. Clastic material including; chert (Ch), dolomite (Cb) and monocrystalline quartz. Bone fragments (B) also present. (G) DMKMM15 (XPL); elongated channel-like voids filled with anhedral calcite (CIV), indicating activity of roots and burrowers towards the top of the North West Remnant. (H) DMKMM15 (XPL); ‘drusy’ sparite directly coating bone fragments, with interlocking calcite crystals within void spaces. Clays, Fe- and Mn- aggregates within groundmass. (I) SSS within DMKMM14 (PPL) towards the top of the North West remnant. Cyclical fining upward trends (white arrows), with hummocky cross stratification preserved at the micro-scale on the right side of the image. (J) Clay crusts (CC) terminated by thin speleothem crusts composed of anhedral calcite (CIV) in sample DMKMM14 (PPL) in SSS.

Microscopically, the fine component of TCB has a well-developed granular microstructure with loose granules (Fig. 12A). However, a dense crumb microstructure consisting of Fe-and Mn- oxide and clay aggregates (Fig. 12B) is also observed towards the base of the Main Makondo profile. Such features likely indicate re-sedimentation of soil material from outside the cavern; or may indicate burrowing or root activity soon after roof collapse (Phillips & FitzPatrick, 1999). Post-depositional illuviation of clay is also indicated by clay coatings observed within pores of TCB (Fig. 12C). Calcite is the most common cement for not only TCB, but for all clastic facies within DMK (Figs. 12A and 12C). The development of strong secondary calcite cement is directly related to the circulation of calcium-carbonate-rich waters percolating through the profile. The most frequent crystalline pattern is mosaic calcite with anhedral crystals which vary greatly in size. Relict crystal boundaries indicating diagenesis of previously precipitated aragonite, or euhedral calcite crystals are also observed (Fig. 12D).

The South East Breccia (Fig. 7) is a spatially distinct feature at DMK separated by several metres from the central talus discussed above (Fig. 4). Here, clastic material overlays wad and flowstone units in contact with the dolomite. Breccia is composed of unsorted, sub-angular dolomite and chert boulders (<30 cm) within a reddish-brown, silty loam matrix. Towards the top of the South East Breccia, sediment is strongly cemented by calcite (Fig. 7). As in dolomite clasts at the base of the Main Makondo, dolomite clasts at the South East Breccia are heavily degraded with a high clay and Fe-oxide content also observed microscopically at crystal boundaries (Fig. 12E). Thus, it is likely that this pinnacle represents a spatially distinct roof collapse within the palaeocave.

### Reworked talus cone breccia and fluvial deposits (SBC)

Breccia units preserved above the TCB in Main and Eastern Makondo, and within the Eastern Remnant, Western and Southern Makondo, Chris’s Gym and the northern exposures of Maddie’s Bath are all characteristic of reworked talus and fluvial deposits and derive from erosion, transport and deposition of karst fill. Thus, collectively these facies are referred to as SBC, however several sub-facies that pertain to distinct phases or to local perturbations of fluvial activity across multiple sections (see Table 1), have also been identified.

**Figure 13:**
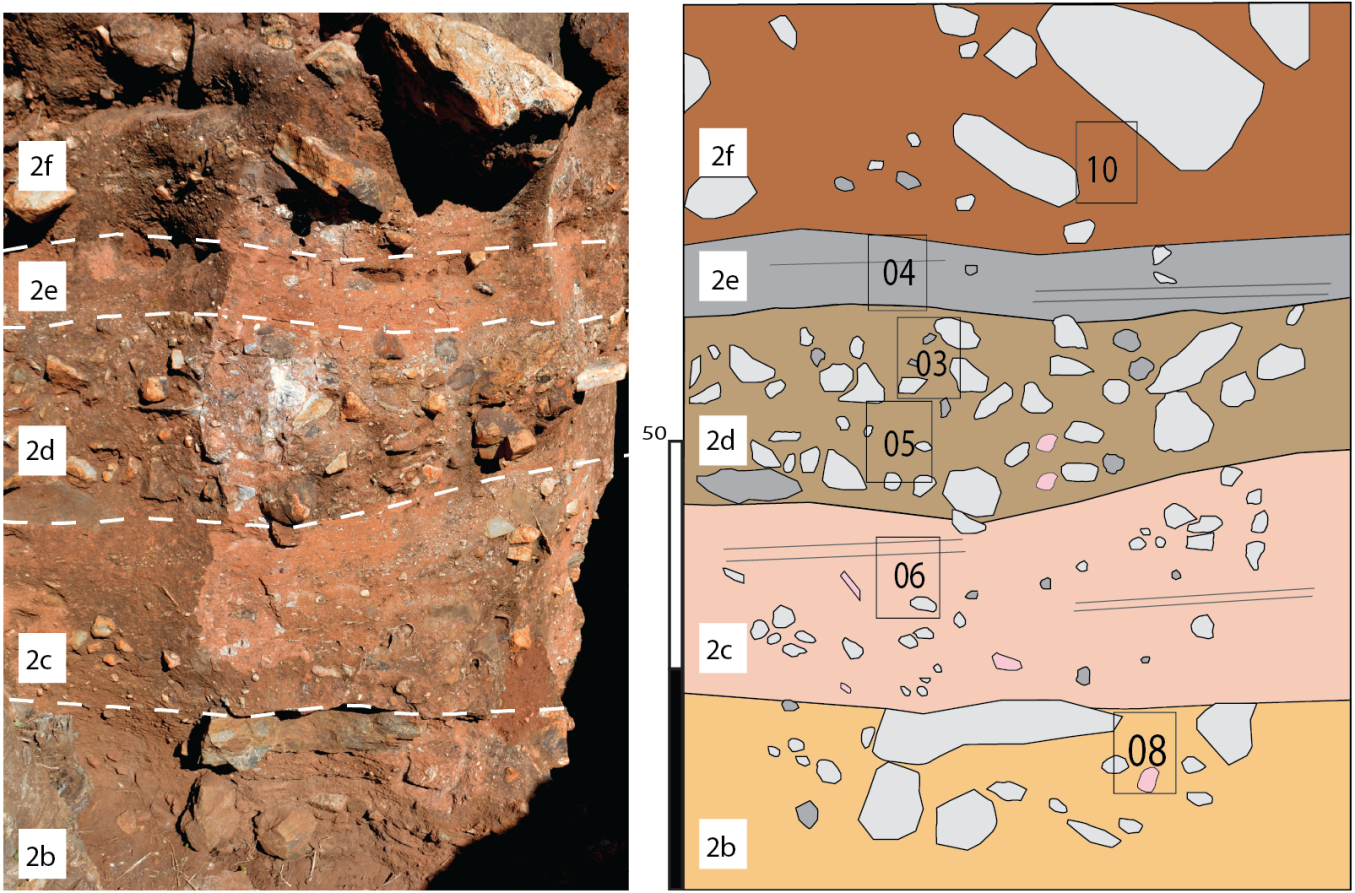
Eastern Remnant. Relationship between SBC sub-facies shown, as well as the location of various micromorphological samples (black boxes).

**Figure 14:**
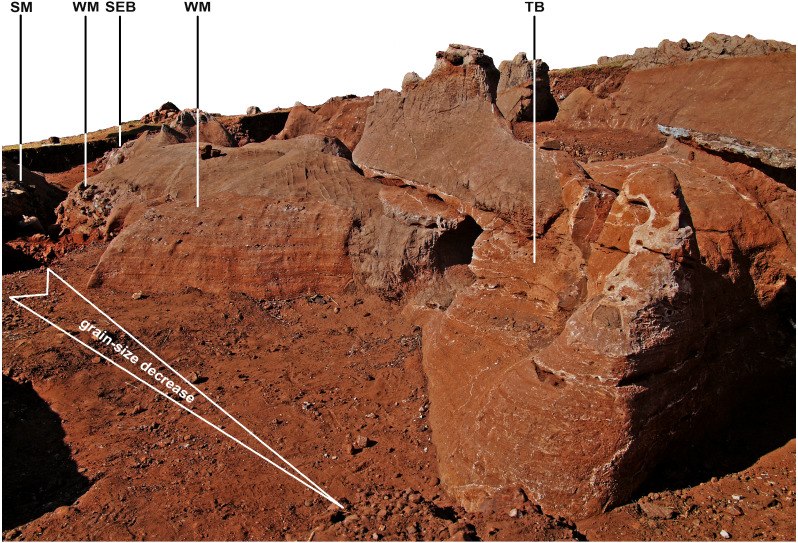
Photogrammetry model of northwest exposures of the DMK deposit, showing grading of siltstone-sandstone facies. WM, Western Makondo (western and eastern extents shown); SM, Southern Makondo; TB, Traynorberg; SEB, South East Breccia.

In general terms, these SBC facies consist of interbedded coarse and fine sub-facies, particularly moving eastward (i.e., Eastern Makondo [Fig. 11] and Eastern Remnant [Fig. 13]). These are best defined following clast size and clast- vs. matrix- supported structure (Table 1). The contacts between sub-facies are typically marked by grain-size changes and are gradual, however shift to subordinately abrupt (*sensu* Catt, 1990). A sharper, erosional upper and lower contact for (2d) is particularly distinct within multiple profiles (Figs. 11 and 13). These represent cyclic phases of moderately high- and low-energy transport. Fluvial activity is also testified by horizontally bedded and imbricated pebble- to cobble-size clasts within coarse SBC units, which derived from the dismantling, transport and redeposition of portions of the talus (Figs. 11 and 13). However, as many of the fossils identified in Eastern Makondo are also articulated (Fig. 5), bones (inc. carcasses) and larger clastic material were not displaced far from the talus. Lateral fining trends indicate fluvial energy decrease westward, which is testified by fine siltstone-sandstone sequences along the far western and north-western extremities of the site (refer to section 4.5 and Fig. 14).

At the micromorphological scale, the composition of the fine fraction of SBC is largely homogenous and consists of silt- to fine sand-sized clasts of monocrystalline quartz, clay aggregates and Fe- and Mn- oxides with a well separated granular microstructure (see Table 2, Fig. 12F). Lastly, the presence of channel voids (Fig. 12G), indicates burrowing or root activity (Stoops, 2003; Stoops, Marcelino & Mees, 2018, p. 402) predominantly within and above sub-facies 2c.

### Siltstone—sandstone facies (SSS)

Finely layered sediments have been previously referred to as siltstone (Keyser & Martini, 1991; Keyser et al., 2000); however, at DMK fine units are characterised by a wider range of grain-sizes from clay to coarse sand, and few gravel sized clasts. The most evident characteristic of these sediments is the cyclic repetition of more or less well-defined fining-upwards sequences, with fine gravel/coarse sand at the base and silt or silty clay at the top. Lenses of medium to fine gravel are sparsely interbedded. Thus, the name ‘siltstone-sandstone’ is more appropriate. At DMK, fine SSS units adhere to the old passage walls and are exposed within and partially around makondo features. SSS deposits also completely infill and surround the Chris’ Gym and occur as small remnants on the wall of the dolomite all along the western, and south-western margin of DMK (Fig. 14). These remnants indicate that a passage that almost exclusively contained SSS deposits ran along the western side of the cave.

SSS units are the distal product of dismantling the talus cone, grading from coarse central breccia deposits (TCB), to mixed coarse (SBC), to fine sediments (SSS). Consequently, the SSS units are also well preserved around the upper exposures of the SBC facies (Fig. 14). Fine lenses of SSS units are well preserved to the west of the site, including patches along the far western wall, Traynorberg, Uluru, Western Remnant and underlying the western flowstone (Figs. 8 and 14). These sediments are relatively homogenous, with only minor changes in grain size, shape and roundness of the clasts, and organised in 0.5–5 cm thick, subhorizontal layers.

The same features observed microscopically within SBC facies are also observed within distal SSS units towards the western exposures of DMK (Table 2). However, differences in clast size and fabric are observed. Clastic material within these units typically includes fine sand- to silt-size, sub-rounded monocrystalline quartz, dolomite and chert granules and angular micaceous fragments. Clay is observed throughout and heavily stained by amorphous Fe-oxides. As with TCB and SBC, SSS units are strongly cemented by calcite. However, micrite and drusy sparite were also observed in SSS units within internal cavities of micro-mammalian bone (Fig. 12H). Intercalations of fining-upwards layers are observed, which are typically terminated by dense, fine silt and clay accumulations sometimes followed by very fine lenses of speleothem (Figs. 12I and 12J). Hummocky cross stratification is also preserved within these SSS units (Fig. 12I), testifying to influxes of water during deposition.

### Organic Components

A primary feature of the central talus cone at DMK, is the abundance of well-preserved partially articulated fossils within SBC, and to a lesser extent, TCB facies. The highest density of these remains has been recovered from a recess between the cave wall and TCB in the NE section of Eastern Makondo, where an entire articulated ribcage of a carnivore was discovered (Fig. 5). Solution pockets within the breccia walls of Main Makondo and Southern Makondo have also yielded articulated bone clusters that are partly included in the decalcified soil, partly within the breccia. Decalcification has only occurred recently, proceeding from the centre of the makondos outwards, thus bones are still well preserved at the edge of makondo features, though are entirely absent in the centre where they have been dissolved (see Fig. 2 in Herries et al., 2018).

**Figure 15:**
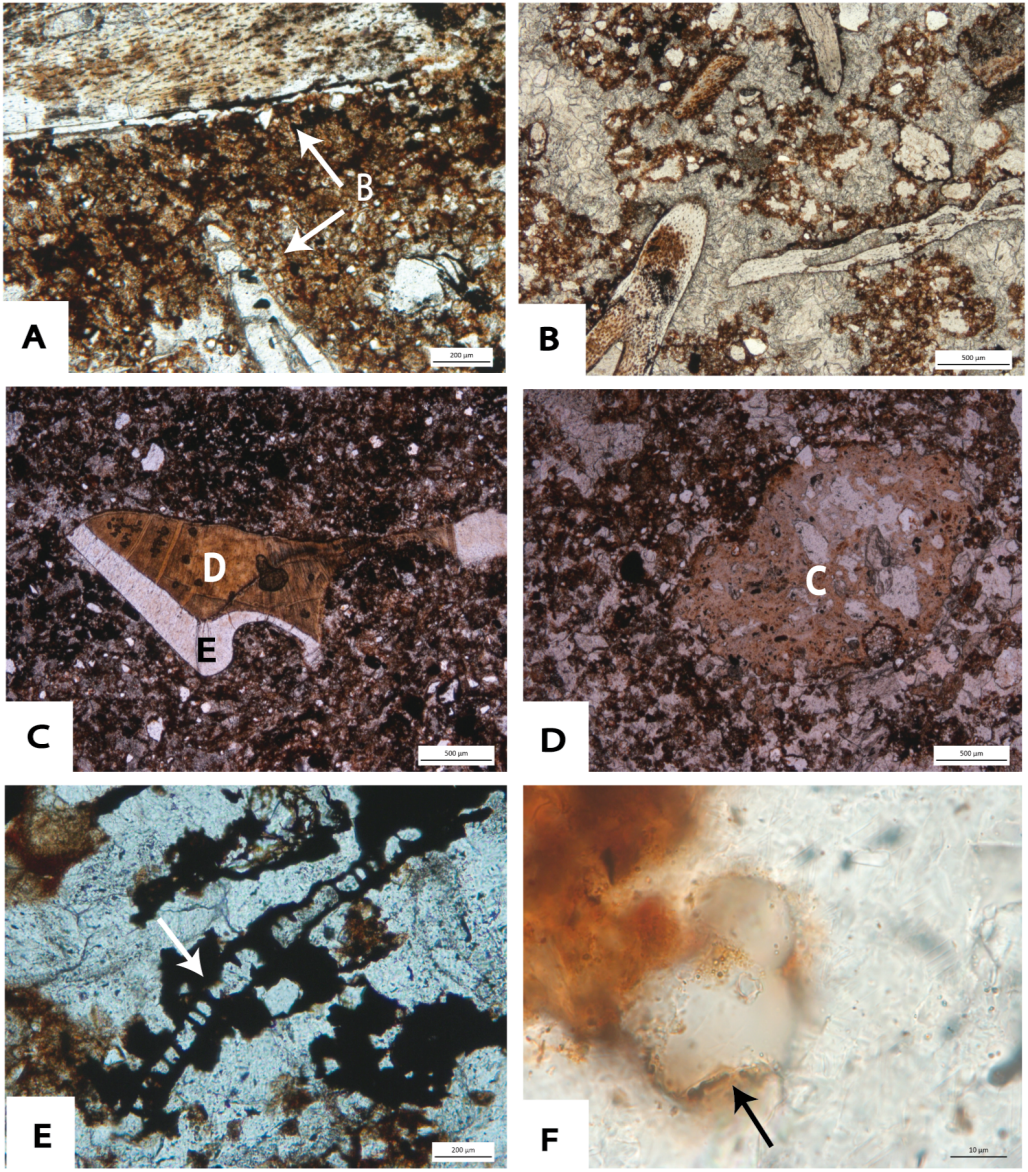
Photomicrographs taken from thin sections at DMK. (A) DMKMM10 (PPL); large bone fragments (B) within a fine groundmass of clays and monocrystalline quartz. (B) DMKMM14 (PPL); dense accumulation of small bone fragments within fine SSS. Complex packing voids with interlocking calcite crystals within. (C) DMKMM07 (PPL); preserved micromammalian tooth fragment including dentine (D) and enamel (E), within a fine sandy loam matrix. (D) DMKMM06 (PPL); Subcircular shaped coprolite (C) with amorphous phosphate composition. (E) DMKMM05 (PPL); woody tissue (red arrow), with a poorly preserved structure. High Mn-O staining on and surrounding fragment. (F) Pollen (black arrow) from DMKMM05 (PPL) on the East Remnant. Form and shape typical of conifer spores from the genus *Podocarpus*.

Macrofossil fragments, partially to completely disarticulated micro- mammalian bone and micro- mammalian teeth, constitute major components of TCB, SBC and distal SSS units at the microscopic scale (Figs. 15A–15C, Table 2). A high density of micro- mammalian fossils is observed within coarser lenses of distal SSS sequences (Fig. 15B). Micro- mammalian dentine and enamel structure was also well preserved and easily identifiable during microscopic analysis (Fig. 15C). Though fossil material is typically larger than the surrounding clasts in SSS, the buoyancy and lower density of bone allowed for transport of partially articulated remains or dense clusters of micro-mammalian fragments during winnowing. Some coprolites, apparently of carnivore, can be easily recognized after their amorphous phosphate composition, shape and occurrence of typical subcircular or teardrop-like voids (Fig. 15D). Fragments which resemble vegetal tissue with poorly preserved cellular structure, were also identified in samples collected from the upper SBC exposures of the Eastern Remnant (Fig. 15E). Significant quantities of pollen were also observed microscopically, providing unique insight into the palaeoenvironment in the CoH. From preliminary analysis, conifer spores pertaining to the genus *Podocarpus* were readily identified (Fig. 15F).

## Discussion

### DMK site formation synthesis

In combining the results of this study with U-Pb, ESR dates and palaeomagnetic data at DMK (Herries et al., 2018), a series of interpretations about the depositional history of the site are presented here. At present, DMK comprises relatively recent elongated karstic dissolution features dissecting dolomite and palaeocave sediments, regardless of their contacts; termed Makondo-karren by Herries & Shaw (2011). These features contain thin palaeocave sediment remnants - breccia (TCB and SBC), siltstone-sandstone (SSS) and flowstone—as well as bulky breccia outcrops in wider passages, testifying to a pre-existing complex cave that was subsequently filled by sediment. Early karstification at DMK commenced with dissolution of dolomite along orthogonal joint sets, which occur at multiple scales at the site and on the surrounding landscape and recur throughout the CoH (Hobbs, 2011). Underground passages with multiple WNW and to a lesser extent, ENE channels have formed along these joints. This elongated shape contrasts with the cavern-like morphology of DMQ (<50 m to the east). At depth, Eastern Makondo also appears to be connected to a deeper cavern that aligns with Porcupine Cave to the south, although the exact point of contact has not been physically reached and is inferred from GPR analysis (Armstrong, 2019). The formation of lower chambers is typical of a multi-phased history of speleogenesis and is characteristic of maze-like caves that forms within complex jointing systems (Stratford, 2011) following fluctuations of the water table. At Sterkfontein, the Name Chamber, situated below the palaeocave deposits of Member 4, contains sediments, archaeological remains and fossils reworked from the higher palaeocave deposits (Stratford et al., 2012). It is likely that dissolution along planar chert beds within the dolomite also formed a continuous chert-roof, fragments of which are partially preserved in the talus breccia. Geological work is supported by ground penetrating radar (GPR) analysis, identifying a series of thick chert layers near DMK (Armstrong, 2019).

**Figure 16:**
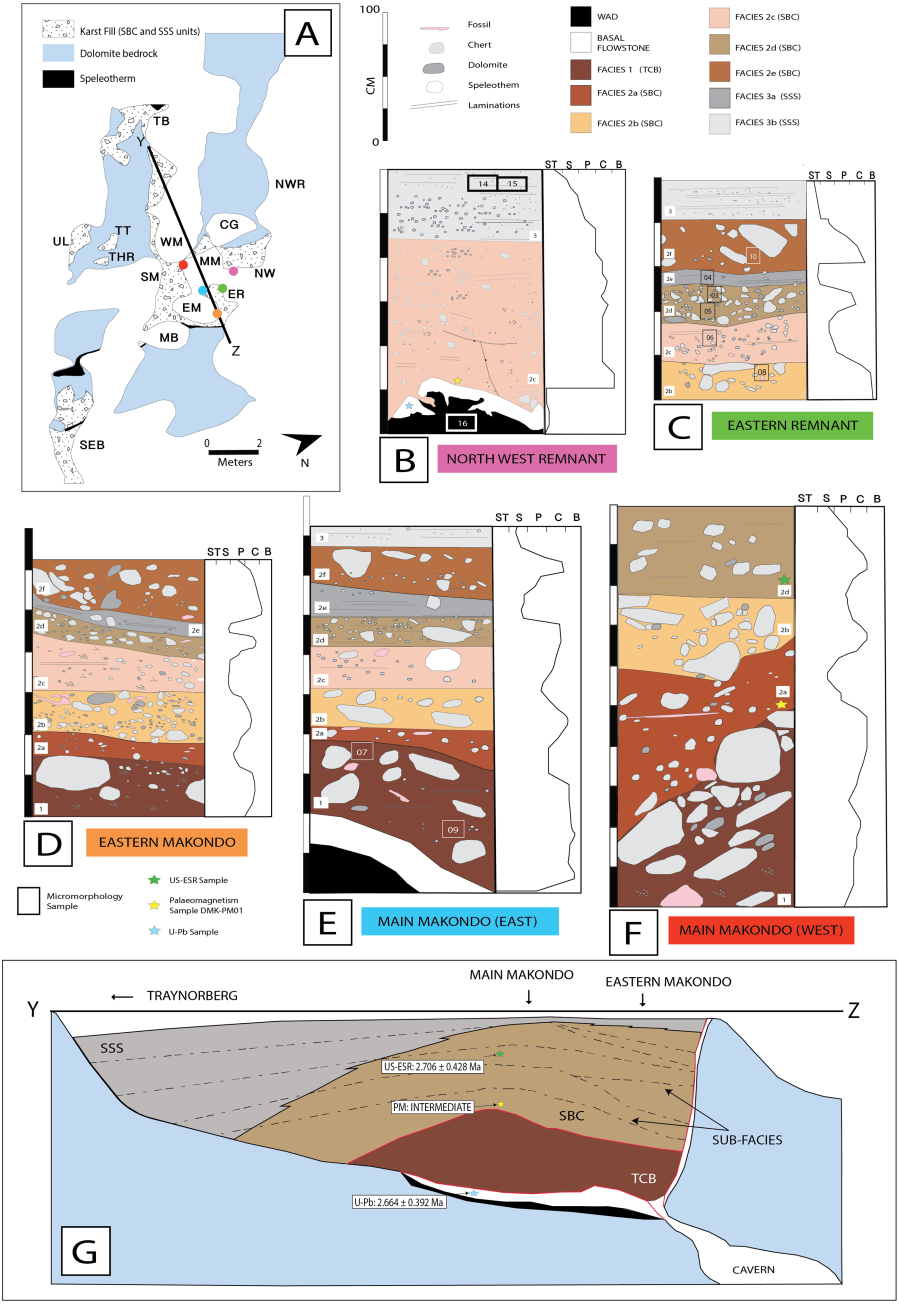
Stratigraphic profiles and interpretations at Drimolen Makondo, focusing on zones proximal to the central talus cone. (A) Simplified plan from Fig. 4. Coloured dots showing location of stratigraphic profiles in B-F, and line (Y–Z) showing orientation of west-to-east depth profile in G. (B–F) Five separate stratigraphic profiles and relative grain size distributions within units identified at various breccia profiles at Makondo. Location of micromorphological samples also shown. (B) Depth profile of the Northwest Remnant, adjacent to the Main Makondo. Exposure showing distal facies of the talus cone, with an overall fining upwards trend. Basal flowstone and wad at the base of this sequence. U-Pb and palaeomagnetic samples taken further north of this exposure. (C) East Remnant, showing SBC subfacies with cyclical fining and coarsening upwards trends. (D) Eastern Makondo showing lateral extent of TCB and SBC subfacies. (E) Main Makondo (eastern wall) adjacent to East Makondo, showing remnant early talus development and basal flowstone and wad formation. (F) Main Makondo (western wall), showing the angle of deposition of early talus cone (NW) and location of ESR and palaeomagnetic samples taken. (G) Simplified schematic showing depth profile (based off Y-Z line in A) displaying allostratigraphic interpretation of the DMK facies. Lateral trends, such as thickening of SBC units to toward East Makondo and fine SSS units towards the top of the sequence due to winnowing of finer fraction from the talus. Dotted lines: Allostratigraphic boundaries mark textural changes. Red line: Erosional surfaces indicating depositional hiatus. Progressive lateral gradation of SBC to SSS facies, largely to the west (towards Traynorberg).

Fe- and Mn- aggregates within basal ‘wad’ formed during late phases of cavern bedrock dissolution. Small amounts of clastic material identified microscopically in wad samples are likely the product of weathering from chert beds interbedded within the dolomite. However, these may have also been introduced during early phases of the cavern breakdown. Water percolation subsequently promoted the precipitation of the basal flowstone, exposed at multiple locations at DMK (Fig. 16), when the communication of the cave with the outside environment was still absent or minor. This is further testified by the lack of clastic material observed microscopically in flowstone samples. Early ceiling breakdown formed the original talus at the interface between the NW Remnant and the entrance of the Main Makondo, accumulating large clasts with dip fabrics to the south-east (including chert, dolomite and shale) at the base of Main Makondo (TCB). In-wash of rubified silty-sandy loam soil sediment formed the talus matrix. This talus is best preserved at the base of the Main Makondo, and to a lesser extent at the base of Eastern Makondo. Its apex was located higher and more to the north, which has been subsequently removed by erosion and dissolution. Consequently, the current exposures show the side of the talus, lithologically overlain by SBC facies, which is synchronously related in an allostratigraphic perspective. Relict crystal boundaries indicating diagenesis of previously precipitated aragonite, or euhedral calcite crystals are observed microscopically, indicating at least two precipitation phases within the TCB facies within Main Makondo. This, along with the sharp upper contact of TCB within the Main Makondo, provide some evidence for an erosional event at the interface between the deposition of TCB and SBC (2a). Lastly, TCB deposits were also identified within the South East Breccia (Fig. 4) and likely represent a spatially distinct, second talus within DMK. The relationship with surrounding SSS facies has yet to be fully established, and therefore temporal associations with the remainder of clastic fill at DMK require further assessment.

The majority of breccia deposits within the central extents of DMK are the product of reworking of the talus cone and fluvial transport within the palaeocave (SBC). Cyclical in-wash events promoted the formation of multiple fining upwards layers within SBC facies, observed within multiple profiles (Figs. 16B–16F). Layers characterised by very poorly sorted medium to fine gravel and somewhat undulating limits, indicate mass transport with high particle concentration, followed by lower-energy and lower-concentration events mobilizing only finer particles –sand or smaller. Cyclical trends are less evident towards the North West Remnant, where a relatively continuous fining upwards pattern is sometimes characterised by scour-and-fill features (Fig. 16B). SBC facies accumulated in the eastern extents of the cavern against the eastern dolomite wall, to which low density carcasses, partially articulated remains and bones were preferentially transported, accumulated and fossilised. This indicates that there was a significantly higher amount of water involved in the processes forming DMK, in comparison to the adjacent DMQ deposit. Partially articulated remains in Eastern Makondo also point to fast carcass transport within the palaeocave, likely with soft tissue still attached during movement. A complete ribcage in the Eastern Makondo gives further evidence to this, as rib-vertebra ligaments typically decay comparatively fast to other components (Nicholson, 1996). Evidence for carnivore presence is also supported by the high density of bone, including those of multiple carnivore taxa (Rovinsky et al., 2015), as well as articulated remains occurring in fine-grained sediments that could not have transported such large bone material. However, a lack of gnawing marks on such material may also indicate that some of the material is a natural death accumulation.

The sedimentologic properties of the SSS facies are similar those of the SBC; the grain-size is however much finer, with fining-upwards layers starting with very fine gravel/coarse sand and typically terminated by dense, fine silt and clay accumulations. These sediments indicate a cyclic medium- to low-energy in-wash within the fluvial depositional environment followed by pooling. Thus, SSS is the product of low-energy erosion and surface transport of the distal talus sides (winnowing: White, 2007), which occurred synchronously with talus formation, though at longer distance and lower particle concentration than SBC facies. SSS sediments are situated in the distal exposures of the cave (concentrated in the western extremities) and overly SBC facies within the central makondo profiles (Fig. 16). The latter likely derived from breccia deposits that were once higher in the sequence, however have since been eroded. Very fine lenses of speleothem sometimes terminate the fining-upwards layers (ref. Figs. 8 and 14), indicating sedimentation stops between flooding phases. The resulting allostratigraphic units are synchronous and lithologically inhomogeneous, grading from proximal coarse SBC to distal fine SSS (Fig. 16G). The boundaries are textural changes that can often be followed through SBC and SSS and may correspond to hiatuses in deposition as well as to erosion surfaces.

Long evolution of clastic facies, as well as of the soils from which they originated, can be inferred by the intense weathering of minerals and rich Fe- and Mn- oxide pedofeatures frequently observed in microscopic analysis throughout multiple sedimentary profiles. The abundance of clay throughout the profile is likely in part due to intense weathering and illuviation caused by prolonged sub-aerial exposure of sediments, though a large portion was inherited by pre-existing soils, eroded and in-washed into the cave. Clay coatings towards the base of the profile are indicative of illuviation through the upper stratigraphic units during flooding or subsequent wet phases (Miedema & Slager, 1972). Due to their infrequent occurrence, it is likely that leaching of clays from higher in the profile was short-lived and hampered by the high pH of the carbonate-rich sediment.

Chronologically, US-ESR, palaeomagnetism and biochronology can be used simultaneously along with U-Pb dates of the basal flowstone to cover different periods of deposition. The basal flowstone is dated to 2.664 ± 0.392 Ma (i.e. forming between 3.035 -2.253 Ma) (Herries et al., 2018; DMK5 in Pickering et al., 2019), providing the maximum age for all clastic deposition and fossil material at DMK. This basal speleothem appears to have a normal magnetic polarity, although weak and as such formed prior to the Gauss-Matuyama Boundary at ∼2.61 Ma (Singer, 2014), suggesting deposition sometime between 3.06 and 2.61 Ma. Based on similar ages retrieved from the basal flowstone at DMQ (2.78–2.61 Ma), the formation of this basal flowstone is likely contemporaneous and offers a maximum age for all vadose palaeokarst within the Drimolen palaeocave system. Flowstones of this age have also been dated at the base of Sterkfontein Member 4 (2.645 ± 0.183 Ma) and Aves Cave at Bolt’s Farm (2.668 ± 0.304 Ma; Pickering et al., 2019), as well as been inferred based on palaeomagnetism capping Member 3 at the Makapansgat Limeworks (Herries & Adams, 2013).

The age of the sediments overlying this flowstone have been defined by a US-ESR age of 2.706 ± 0.428 Ma on a bovid tooth towards the top of the talus breccia (i.e., SBC [2c]: Figs. 16D–16E) (Herries et al., 2018); indicating an age sometime between 3.06 and 2.28 Ma when combining U-Pb ages (Herries et al., 2018). Coupling of US-ESR and U-Pb ages at DMK is insufficient to give well bracketed ages; however, dates can be further constrained through palaeomagnetism. Samples taken from various locations in the clastic infill, show a distinct palaeomagnetism trend during deposition. DMK-PM03 overlying the basal flowstone returns both normal (VGP; 46.5°) and intermediate polarities (VGP; −7.3°); DMK-PM01 within finer clastic lenses of SBC also records intermediate polarities (VGP; −2.8°); and sample DMK-PM04 towards the top of the sequence at Main Makondo records a reversed polarity (VGP; −79.9°) (Herries et al., 2018). This data is consistent with a change from normal to reversed polarity occurring at the Gauss-Matuyama Boundary at 2.61 Ma, consistent with the age estimates from US-ESR and U-Pb (3.04–2.28 Ma). Though not a reliable stand-alone method, biochronology is also a useful tool in this case. The *Metridiochoerus* specimen (DNM 57) located at DMK, which likely derived from a Stage 1 *Metridiochoerus andrewsi* has only been found in sites pre- 2.61 Ma (e.g., Makapansgat Limeworks between 3.03 and 2.61 Ma; Rovinsky et al., 2015; Herries & Adams, 2013; Herries et al., 2018); further accompanying evidence for the ∼2.61 Ma age.

These conclusions are supported in sedimentological and micromorphological analysis of DMK, as the granular microstructure is consistently well-separated within breccia deposits. This can be associated with a relatively fast deposition, which was sequentially followed by rapid cementation. The remains of a large partially articulated ribcage in Eastern Makondo corroborate this hypothesis. Although less absolute, a smaller cavern morphology can also imply a shorter deposition history. The small size of the talus cone, chert beds (i.e., cavern ceiling) directly adjacent to the deposit, bioturbation and/or root penetration, and pollen observed microscopically within multiple profiles, can all be associated with a shallower chamber. Thus, a relatively short infilling time-span centred on 2.61 Ma is presented.

### Broader significance of DMK

DMK is a rare South African example of a Pliocene-Pleistocene boundary aged fossil site. This time period in which DMK is set (∼2.61 Ma) is palaeoenvironmentally significant, as it aligns with the onset of a long-term trend toward aridification in eastern Africa (De Menocal, 2011) and more variable climatic conditions. This aligns with notable first appearance and extinction events and behavioural milestones in eastern Africa clustered between the 2.9–2.6 Ma; including, the extinction of *Australopithecus afarensis*, the emergence of robust- australopiths *Paranthropus*, and larger brained *Homo* lineage sometime after 2.7 Ma (De Menocal, 2011; Villmoare et al., 2015). Changes in faunal species have also been documented during this time frame, with increased number of grazing bovid species, supporting views that climatic aridity and variability <3 Ma led to ecological shifts in Africa (Cerling, 1992).

In contrast the ∼2.61 Ma age range for DMK deposits places it within a time-period in southern Africa where there are few fossil sites and little palaeoenvironmental information. Some climatic data suggests that major climate change occurred in South Africa slightly later than eastern Africa, with major changes occurring between ∼2.3–2.0 Ma (De Menocal, 2011; Herries et al., 2020; Kimbel, 2009; Maslin & Trauth, 2009; Potts, 1998). This correlates with the extinction of *Australopithecus* and the first occurrence of *Paranthropus robustus*, *Homo erectus* and archaeology at DMQ, and perhaps also Swartkrans Member 1 Lower Bank (Gibbon et al., 2014; Herries et al., 2020). Deposits dated to ≥ 2.61 Ma include the *Australopithecus africanus* bearing Makapangsat Limeworks Member 3 and the PCS deposits of the Buxton- Norlim Limeworks Pinnacles Type Site (Taung: 3.03–2.58 Ma) (Herries & Adams, 2013), both of which are outside Gauteng Province in the CoH; the non-hominin bearing fossil site of Hoogland at 3.11–2.61 Ma (Adams et al., 2010), and potentially the australopith bearing Sterkfontein Members 2 and 4 (between ∼3.7–2.2 Ma [Pickering & Kramers, 2010; Herries & Shaw, 2011; Kramers & Dirks, 2017a; Kramers & Dirks, 2017b; Granger et al., 2015; Stratford et al., 2017] and 2.61 to 2.07 Ma respectively [Pickering et al., 2019]). Therefore, with the exception of Sterkfontein and Malapa, DMK is a rare example of an australopithecine-aged site within the Gauteng Province in the CoH. The level of erosion at DMK may provide further insight into this, as exposure and ongoing deterioration of Pliocene sites has since caused their complete removal from the current land surface. In addition, assessments at DMK have uncovered a unique accumulation of faunal remains (Rovinsky et al., 2015; Herries et al., 2018); as well as coprolites, pollen and potential vegetal tissue as part of this study, and vegetal tissue at DMQ (Herries et al., 2020). This is comparable to isolate findings of a pollen and wood tissue fragments within a coprolite identified at Malapa (Bamford et al., 2010), and woody tissue fragments identified at Sterkfontein Member 4 (Bamford, 1999). First, analysis of pollen and phytoliths at Malapa corresponds to *Podocarpus* pollen identified at DMK. The genus *Podocarpus* has also been identified in marine sediments at ∼2 Ma off the Namibian coast (Dupont, 2006). In these cases, both pollen and microbotanical remains correspond to the presence of conifer trees on the landscape from the terminal Pliocene to Early Pleistocene. Today this species is found in the greatest abundance in KwaZulu-Natal and the Eastern Cape of South Africa (Adie & Lawes, 2011). The modern forms are interpreted as temperate forest remnants that would have dominated during glacial periods and retreated to more montane environments during interglacials (Adie & Lawes, 2011). From analysis of woody tissue samples at Sterkfontein Member 4, which is of comparable age to DMK, *Anastrabe integerrima* and *Dichapetalum mombuttense* samples were also identified (Bamford, 1999). These morphotypes do not contradict interpretations at DMK or Malapa, however all collectively support the presence of gallery forest and higher rainfall than present day within this region. However, as there are major differences between fossil taxa at DMK and DMQ (Adams et al., 2016; Rovinsky et al., 2015), further analysis of palaeoecological material at the site is still required. In addition to this, as DMK has significant quantities of pollen preserved, a comprehensive palynological assessment is now obtainable. With this analysis and supporting geological data from the site, palaeoenvironmental reconstructions from the terminal Pliocene to Early Pleistocene are also achievable and a focus during ongoing assessment.

## Conclusions

New advances in chronological methods have greatly aided contextualising notoriously complex karst deposits in southern Africa, however their application can only be effective with a comprehensive understanding of a site’s stratigraphy. In combining litho- and allostratigraphy, micromorphology and geochronology, this model deviates from the classic ‘Member System’ and has been effective in assessing the rapid depositional history at DMK. The importance in undertaking such a study at DMK is multifaceted. The first is that it provides an opportunity to assess an ancient palaeocave with intact stratigraphies, as the site has not undergone extensive speleothem mining. This has made stratigraphic interpretations more accessible, as multiple sequences within the site remain *in situ*. The richness in fossil remains including articulated fossils, coprolites and micro-mammals are also unique, and well-preserved in both the breccia facies and within the decalcified material. Lastly, DMK is now well confined as an australopithecine-aged site (∼2.61 Ma) that correlates with an ecologically significant time period associated with long-term aridification and variable climatic events, as well as first appearance and extinction of species. The high percentage of faunal remains as well as the microscopic observation of pollen grains, indicates DMK is a significant site for ongoing palaeoenvironmental studies.

## Additional Information and Declarations

### Field Study Permissions

The following information was supplied relating to field study approvals (i.e., approving body and any reference numbers): Khethi Nkosi, the landowner at Drimolen, granted permission to work at the site in conjunction with the South African Heritage Resources Agency (SAHRA) (Permit ID 2035).

### Data Availability

The following information was supplied regarding data availability: Photographs of Drimolen Makondo are available in the Figures and field observations are available in Tables 1 and 2.

## References

[ref-1] Adams JW, Herries AIR, Hemingway J, Kegly A, Kgazi L, Hopley P, Reede H, Potze S, Thackeray F (2010). Initial fossil discoveries from Hoogland, a new Pliocene primate-bearing karstic system in Gauteng Province, South Africa. Journal of Human Evolution.

[ref-2] Adams JW, Rovinsky DS, Herries AI, Menter CG (2016). Macromammalian faunas, biochronology and palaeoecology of the early Pleistocene Main Quarry hominin-bearing deposits of the Drimolen Palaeocave System, South Africa. PeerJ.

[ref-3] Adie H, Lawes MJ, Turner BL, Cernusak LA (2011). Podocarps in Africa: temperate zone relicts or rainforest survivors?. Ecology of the Podocarpaceae in Tropical Forests.

[ref-4] Armstrong BJ (2019). Application of new 3D geospatial and geophysical techniques to map, analyse and interpret surface and subsurface deposits in fossil bearing palaeocaves (UNESCO Cradle of Humankind, South Africa). Unpublished (PhD) Thesis.

[ref-5] Bamford M (1999). Pliocene fossil woods from an early hominid cave deposit, Sterkfontein, South Africa. South African Journal of Science.

[ref-6] Bamford MK, Neumann FH, Pereira LM, Scott L, Dirks PHGM, Berger LR (2010). Botanical remains from a coprolite from the Pleistocene hominin site of Malapa, Sterkfontein Valley, South Africa. Palaeontologia Africana.

[ref-7] Berger LR, De Ruiter DJ, Churchill SE, Schmid P, Carlson KJ, Dirks PH, Kibii JM (2010). Australopithecus sediba: a new species of Homo-like australopith from South Africa. Science.

[ref-8] Berger LR, Hawks J, De Ruiter DJ, Churchill SE, Schmid P, Delezene LK, Kivell TL, Garvin HM, Williams SA, DeSilva JM, Skinner MM (2015). Homo naledi, a new species of the genus Homo from the Dinaledi Chamber, South Africa. Elife.

[ref-9] Bosák P, Ford DC, Glazek J, Horácek I (2015). Paleokarst: a systematic and regional review.

[ref-10] Boschian G (1997). Sedimentology and soil micromorphology of the late Pleistocene and early Holocene deposits of Grotta dell’Edera (Trieste Karst, NE Italy). Geoarchaeology: An International Journal.

[ref-11] Bountalis AC, Kuhn BF (2014). Cave usage by multiple taphonomic agents: issues towards interpreting the fossil bearing cave deposits in South Africa. American Journal of Zoological Research.

[ref-12] Braga J, Fourvel JB, Lans B, Bruxelles L, Thackeray JF, Braga J, Thackeray JF (2016). Evolutionary, chrono-cultural and palaeoenvironmental backgrounds to the Kromdraai site: a regional perspective. Kromdraai: A Birthplace of Paranthropus in the Cradle of Humankind.

[ref-13] Brain CK (1976). A re-interpretation of the Swartkrans Site and it remains. South African Journal of Science.

[ref-14] Brain CK (1981). The hunters or the hunted? An introduction to African Cave Taphonomy.

[ref-15] Brink ABA, Partridge TC (1980). The nature and genesis of solution cavities (makondos) in Transvaal cave breccia. Palaeontologica Africana.

[ref-16] Broom R (1938). The pleistocene anthropoid apes of South Africa. Nature.

[ref-17] Bruxelles L, Clarke RJ, Maire R, Ortega R, Stratford D (2014). Stratigraphic analysis of the Sterkfontein StW 573 Australopithecus skeleton and implications for its age. Journal of Human Evolution.

[ref-18] Bruxelles L, Marie R, Couzens R, Thackeray JF, Braga J, Braga T (2016). A revised stratigraphy of Kromdraai. Kromdraai: a Birthplace of *Paranthropus* in the Cradle of Humankind.

[ref-19] Bullock P, Thompson ML (1985). Micromorphology of alfisols. Soil micromorphology and soil classification.

[ref-20] Butzer KW (1976). Lithostratigraphy of the Swartkrans Formation. South African Journal of Science.

[ref-21] Caruana MV (2017). Lithic production strategies in the Oldowan assemblages from Sterkfontein member 5 and Swartkrans member 1, Gauteng province, South Africa. Journal of African Archaeology.

[ref-22] Catt JA (1990). Paleopedology manual.

[ref-23] Cerling TE (1992). Development of grasslands and savannas in East Africa during the Neogene. Palaeogeography, Palaeoclimatology, Palaeoecology.

[ref-24] Clarke RJ (2019). Excavation, reconstruction and taphonomy of the StW 573 Australopithecus prometheus skeleton from Sterkfontein Caves, South Africa. Journal of Human Evolution.

[ref-25] Courty MA, Goldberg P, Macphail R (1989). Soils and micromorphology in archaeology.

[ref-26] Dart RA (1925). Australopithecus africanus the man-ape of South Africa. Nature.

[ref-27] Davis NK, McMillan BA (2017). Geology of quartz-lined hypogene caves of Southeastern Arizona. Klimchouk others, Hypogene Karst Regions and Caves of the World.

[ref-28] De Menocal PB (2011). Climate and human evolution. Science.

[ref-29] Dirks PH, Berger LR (2013). Hominin-bearing caves and landscape dynamics in the Cradle of Humankind, South Africa. Journal of African Earth Sciences.

[ref-30] Dirks PH, Kibii JM, Kuhn BF, Steininger C, Churchill SE, Kramers JD, Pickering R, Farber DL, Mériaux AS, Herries AI, King GC (2010). Geological setting and age of Australopithecus sediba from southern Africa. Science.

[ref-31] Dirks PH, Placzek CJ, Fink D, Dosseto A, Roberts E (2016). Using 10Be cosmogenic isotopes to estimate erosion rates and landscape changes during the Plio-Pleistocene in the Cradle of Humankind, South Africa. Journal of Human Evolution.

[ref-32] Dirks PH, Roberts EM, Hilbert-Wolf H, Kramers JD, Hawks J, Dosseto A, Duval M, Elliott M, Evans M, Grün R, Hellstrom J (2017). The age of Homo Naledi and associated sediments in the rising Star Cave, South Africa. elife.

[ref-33] Dubois C, Quinif Y, Baele JM, Barriquand L, Bini A, Bruxelles L, Dandurand G, Havron C, Kaufmann O, Lans B, Maire R (2014). The process of ghost-rock karstification and its role in the formation of cave systems. Earth-Science Reviews.

[ref-34] Dupont LM (2006). Late Pliocene vegetation and climate in Namibia (southern Africa) derived from palynology of ODP Site 1082. Geochemistry, Geophysics and Geosystems.

[ref-35] Eriksson PG, Reczko BFF (1995). The sedimentary and tectonic setting of the Transvaal to the Bushveld complex. Journal of African Earth Science.

[ref-36] FitzPatrick EA (1984). Micromorphology of soils.

[ref-37] Forbes MS, Bestland EA (2007). Origin of the sedimentary deposits of the Naracoorte Caves, South Australia. Geomorphology.

[ref-38] Ford DC (1995). Paleokarst as a target for modern karstification. Carbonates and Evaporites.

[ref-39] Ford DC, Williams PW (1989). Karst geomorphology and hydrology.

[ref-40] Ford D, Williams PD (2013). Karst Hydrology and Geomorphology.

[ref-41] Gibbon RJ, Pickering TR, Sutton MB, Heaton JL, Kuman K, Clarke RJ, Brain CK, Granger DE (2014). Cosmogenic nuclide burial dating of hominin-bearing Pleistocene cave deposits at Swartkrans, South Africa. Quaternary Geochronology.

[ref-42] Goldberg P (2000). Micromorphology and site formation at Die Kelders cave I, South Africa. Journal of Human Evolution.

[ref-43] Goldberg P (2001). Some micromorphological aspects of prehistoric cave deposits. Cahiers d’Archéologie du CELAT.

[ref-44] Goldberg P, Bar-Yosef O (2002). Site formation processes in Kebara and Hayonim caves and their significance in Levantine prehistoric caves. Neandertals and modern humans in Western Asia.

[ref-45] Goldberg P, Berna F (2010). Micromorphology and context. Quaternary International.

[ref-46] Granger DE, Gibbon RJ, Kuman K, Clarke RJ, Bruxelles L, Caffee MW (2015). New cosmogenic burial ages for Sterkfontein Member 2 Australopithecus and Member 5 oldowan. Nature.

[ref-47] Herries AIR (2003). Magnetostratigraphic Seriation of South African Hominin Palaeocaves. Geomagnetism Laboratory, Department of Archaeology.

[ref-48] Herries AIR, Adams JW (2013). Clarifying the context, dating and age range of the Gondolin hominins and *Paranthropus* in South Africa. Journal of Human Evolution.

[ref-49] Herries AIR, Adams JW, Joannes-Boyau R, Armstrong B, Baker S, Blackwood AF, Boshian G, Caruana MV, Penzo-Kajewski P, Murszewski A, Rovinsky DS (2019). Integrating palaeocaves into palaeolandscapes: An analysis of cave levels and karstification history across the Gauteng Malmani dolomite, South Africa. Quaternary Science Reviews.

[ref-50] Herries AIR, Curnoe D, Adams JW (2009). A multi-disciplinary seriation of early Homo and Paranthropus bearing palaeocaves in southern Africa. Quaternary International.

[ref-51] Herries AIR, Martin JM, Leece AB, Adams JW, Boschian G, Joannes-Boyau R, Edwards TR, Mallett T, Massey J, Murszewski A, Neubauer S, Pickering R, Strait D, Armstrong BJ, Baker S, Caruana MV, Denham T, Hellstrom J, Moggi-Cecchi J, Mokobane S, Penzo-Kajewski P, Rovinsky DS, Schwartz GT, Stammers RC, Wilson C, Woodhead J, Menter C (2020). Contemporaneity of *Australopithecus*, Paranthropus and early *Homo erectus* in S. Africa. Science.

[ref-52] Herries AIR, Murszewski A, Pickering R, Mallett T, Joannes Boyau R, Armstrong B, Adams JW, Baker S, Blackwood AF, Penzo Kajewski P, Kappen P, Leece AB, Martin J, Rovinsky D, Boschian G (2018). Geoarchaeological and 3D visualisation approaches for contextualising in-situ fossil bearing palaeokarst in South Africa: a case study from the ∼2.61 Ma Drimolen Makondo. Quaternary International.

[ref-53] Herries AIR, Pickering R, Adams JW, Curnoe D, Warr G, Latham AG, Shaw J, Reed KE, Fleagle JG, Leakey R (2013). A multi-disciplinary perspective on the age of *Australopithecus* in Southern Africa. The Paleobiology of Australopithecus.

[ref-54] Herries AIR, Reed KE, Kuykendall KL, Latham AG (2006). Speleology and magnetobiostratigraphic chronology of the Buffalo Cave fossil site, Makapansgat, South Africa. Quaternary Research.

[ref-55] Herries AIR, Shaw J (2011). Palaeomagnetic analysis of the Sterkfontein palaeocave deposits: implications for the age of the hominin fossils and stone tool industries. Journal of Human Evolution.

[ref-56] Hobbs PJ (2011). Situation assessment of the surface water and groundwater resource environements in the Cradle of Humankind World Heritage Area. Report prepared for the Management Authority. Department of Economic Development, Gauteng Province. South Africa.

[ref-57] Karkanas P, Bar-Yosef O, Goldberg P, Weiner S (2000). Diagenesis in prehistoric caves: the use of minerals that form in situ to assess the completeness of the archaeological record. Journal of Archaeological Science.

[ref-58] Karkanas P, Goldberg P (2010). Site formation processes at Pinnacle point Cave 13B (Mossel Bay, Western Cape Province, South Africa): resolving stratigraphic and depositional complexities with micromorphology. Journal of Human Evolution.

[ref-59] Karkanas P, Goldberg P, Shroder J, Frumkin S (2013). Micromorphology of cave sediments. Treatise on Geomorphology.

[ref-60] Karkanas P, Kyparissi-Apostolika N, Bar-Yosef O, Weiner S (1999). Mineral assemblages in Theopetra, Greece: a framework for understanding diagenesis in a prehistoric cave. Journal of Archaeological Science.

[ref-61] Keyser AW, Martini J (1991). Haasgat: a new Plio-Pleistocene fossil occurrence. Palaeoecology of Africa.

[ref-62] Keyser AW, Menter CG, Moggi-Cecchi J, Pickering TR, Berger LR (2000). Drimolen: a new hominid-bearing site in Gauteng, South Africa. South African Journal of Science.

[ref-63] Kimbel WH, Grine FE, Fleagle JG, Leakey REF (2009). The origin of *Homo*. In the first humans—origin and early evolution of the genus homo.

[ref-64] Kramers JD, Dirks PH (2017a). The age of fossil StW573 (‘Little Foot’): an alternative interpretation of 26Al/10Be burial data. South African Journal of Science.

[ref-65] Kramers JD, Dirks PH (2017b). The age of fossil StW573 (‘Little Foot’): Reply to comments by Stratford others. South African Journal of Science.

[ref-66] Latham AG, Herries AI, Kuykendall K (2003). The formation and sedimentary in- filling of the Limeworks Cave, Makapansgat, South Africa.

[ref-67] Latham AG, Herries A, Quinney P, Sinclair A, Kuykendall K (1999). The Makapansgat australopithecine site from a speleological perspective. Geological Society, London, Special Publications.

[ref-68] Latham AG, McKee JK, Tobias PV (2007). Bone breccias, bone dumps, and sedimentary sequences of the western Limeworks, Makapansgat, South Africa. Journal of Human Evolution.

[ref-69] Macphail RI, Goldberg P, Linderholm J, Stringer CB, Barton RNE, Finlayson JC (2000). Geoarchaeological investigation of sediments from Gorham’s and Vanguard Caves, Gibraltar: microstratigraphical (soil micromorphological and chemical) signatures. Neanderthals on the Edge.

[ref-70] Martini J, Kavalieris I (1976). The karst of Transvaal (South Africa). International Journal of Speleology.

[ref-71] Maslin MA, Trauth MH (2009). Plio-Pleistocene East African pulsed climate variability and its influence on early human evolution. The First Humans–Origin and Early Evolution of the Genus Homo.

[ref-72] Martini JE, Wipplinger PE, Moen HF, Keyser A (2003). Contribution to the speleology of Sterkfontein cave, Gauteng Province, South Africa. International Journal of Speleology.

[ref-73] Menter CG, Kuykendall KL, Keyser AW, Conroy GC (1999). First record of hominid teeth from the Plio-Pleistocene site of Gondolin, South Africa. Journal of Human Evolution.

[ref-74] Miedema R, Slager S (1972). Micromorphological quantification of clay illuviation. Journal of Soil Science.

[ref-75] Murszewski A, Edwards TR, Cruden AR, Armstrong B, Boschian G, Herries AI (2019). Regional geological formation and speleogenesis of the ‘Fossil Hominid Sites of South Africa ’UNESCO World Heritage Site. Earth-Science Reviews.

[ref-76] Ngoloyi NM, Dumoncel J, Thackeray JF, Braga J (2020). A new method to evaluate 3D spatial patterns within early hominin-bearing sites. An example from Kromdraai (Gauteng Province, South Africa). Journal of Archaeological Science: Reports.

[ref-77] Nicholson RA (1996). Bone degradation, burial medium and species representation: debunking the myths, an experiment-based approach. Journal of Archaeological Science.

[ref-78] Nicosia C, Stoops G (2017). Archaeological soil and sediment micromorphology.

[ref-79] North American Commission on Stratigraphic Nomenclature (2005). North American stratigraphic code. AAPG Bulletin.

[ref-80] Osborne RAL (1999). The inception horizon hypothesis in vertical to steeply-dipping limestone: applications in New South Wales, Australia. Cave and Karst Science.

[ref-81] Osborne RAL (2007). Cathedral Cave, Wellington Caves, New South Wales, Australia. A multiphase, non-fluvial cave. Earth Surface Processes and Landforms: The Journal of the British Geomorphological Research Group.

[ref-82] Partridge TC (1973). Geomorphological dating of the cave opening at Makapansgat, Sterkfontein, Swartkrans and Taung. Nature.

[ref-83] Partridge TC (1978). Re-appraisal of lithostratigraphy of Sterkfontein hominid site. Nature.

[ref-84] Partridge TC (1979). Re-appraisal of lithostratigraphy of Makapansgat Limeworks hominid site. Nature.

[ref-85] Partridge TC, de Lumley H, de Lumley MA (1982). L’Homo erectus et la place de l’homme de Tautavel parmi les hominidés fossiles. Congrés international de paléontologie humaine, ler Congrés.

[ref-86] Partridge TC (2000). Hominid-bearing cave and tufa deposits. Oxford Monographs on Geology and Geophysics.

[ref-87] Partridge TC, Granger DE, Caffee MW, Clarke RJ (2003). Lower Pliocene hominid remains from Sterkfontein. Science.

[ref-88] Partridge TC, Watt IB (1991). The stratigraphy of the Sterkfontein hominid deposit and its relationship to the underground cave system. Palaeontology Africa.

[ref-89] Phillips DH, FitzPatrick EA (1999). Biological influences on the morphology and micromorphology of selected Podzols (Spodosols) and Cambisols (Inceptisols) from the eastern United States and north-east Scotland. Geoderma.

[ref-90] Pickering R, Hancox PJ, Lee-Thorp JA, Grün R, Mortimer GE, McCulloch M, Berger LR (2007). Stratigraphy, U-Th chronology, and paleoenvironments at Gladysvale Cave: insights into the climatic control of South African hominin-bearing cave deposits. Journal of Human Evolution. African Paleoclimate and Human Evolution.

[ref-91] Pickering R, Herries AI, Woodhead JD, Hellstrom JC, Green HE, Paul B, Ritzman T, Strait DS, Schoville BJ, Hancox PJ (2019). U–Pb-dated flowstones restrict South African early hominin record to dry climate phases. Nature.

[ref-92] Pickering R, Kramers JD (2010). Re-appraisal of the stratigraphy and determination of new U-Pb dates for the Sterkfontein hominin site, South Africa. Journal of Human Evolution.

[ref-93] Pickering R, Kramers JD, Hancox PJ, De Ruiter DJ, Woodhead JD (2011b). Contemporary flowstone development links early hominin bearing cave deposits in South Africa. Earth and Planetary Science Letters.

[ref-94] Potts R (1998). Environmental hypotheses of hominin evolution. American Journal of Physical Anthropology: The Official Publication of the American Association of Physical Anthropologists.

[ref-95] Reynolds SC, Clarke RJ, Kuman KA (2007). The view from the Lincoln Cave: mid to late-Pleistocene fossil deposits from Sterkfontein hominid site, South Africa. Journal of Human Evolution.

[ref-96] Reynolds SC, Vogel JC, Clarke RJ, Kuman KA (2003). Preliminary results of excavations at Lincoln Cave, Sterkfontein, South Africa. South African Journal of Science.

[ref-97] Rovinsky DS, Herries AI, Menter CG, Adams JW (2015). First description of in situ primate and faunal remains from the Plio-Pleistocene Drimolen Makondo palaeocave infill, Gauteng, South Africa. Palaeontologia Electronica.

[ref-98] Schwartz RN, Ziari M, Trivedi S (1994). Electron paramagnetic resonance and an optical investigation of photorefractive vanadium-doped CdTe. Physical Review B.

[ref-99] Singer BS (2014). A Quaternary geomagnetic instability time scale. Quaternary Geochronology.

[ref-100] Stammers RC, Caruana MV, Herries AI (2018). The first bone tools from Kromdraai and stone tools from Drimolen, and the place of bone tools in the South African Earlier Stone Age. Quaternary International.

[ref-101] Stoops G (2003). Guidelines for analysis and description of soil and regolith thin sections.

[ref-102] Stoops G, Marcelino V, Mees F (2018). Interpretation of micromorphological features of soils and regoliths.

[ref-103] Stratford DJ (2011). The underground central deposits of the Sterkfontein Caves, South Africa. Doctoral dissertation.

[ref-104] Stratford DJ, Bruxelles L, Clarke RJ, Kuman K (2012). New stratigraphic interpretations of the fossil and artefact-bearing deposits of the Name Chamber, Sterkfontein. South African Archaeological Bulletin.

[ref-105] Stratford DJ, Grab S, Pickering TR (2014). The stratigraphy and formation history of fossil- and artefact-bearing sediments in the Milner Hall, Sterkfontein Cave, South Africa: new interpretations and implications for palaeoanthropology and archaeology. Journal of African Earth Science.

[ref-106] Stratford D, Granger DL, Bruxelles L, Clarke RJ, Kuman K, Gibbon RJ (2017). Comments on ‘The age of fossil StW573 (‘Little Foot’): an alternative interpretation of 26Al/10Be burial data’. South African Journal of Science.

[ref-107] Stratford D, Palmer N, Klimchouk A, A DE, Waele JS, Auler A, Audra P (2017). A review of the geomorphological context and stratigraphy of the sterkfontein caves, South Africa. Hypogene Karst Regions and caves of the world.

[ref-108] Val A, Dirks PH, Backwell LR, D’Errico F, Berger LR (2015). Taphonomic analysis of the faunal assemblage associated with the hominins (Australopithecus sediba) from the Early Pleistocene cave deposits of Malapa, South Africa. PLOS ONE.

[ref-109] Villmoare B, Kimbel WH, Seyoum C, Campisano CJ, DiMaggio EN, Rowan J, Braun DR, Arrowsmith JR, Reed KE (2015). Early Homo at 2.8 Ma from Ledi-Geraru, Afar, Ethiopia. Science.

[ref-110] Ward I, Winter S, Dotte-Sarout E (2016). The Lost Art of Stratigraphy? A consideration of excavation strategies in Australian Indigenous archaeology. Australian Archaeology.

[ref-111] Wenk HR, Bulakh A (2004). Minerals: their constitution and origin.

[ref-112] White WB (2007). Cave sediments and palaeoclimate. Journal of Cave and Karst Studies.

[ref-113] Wilkinson MJ, Tobias PV (1985). Lower-lying and possibly older fossiliferous deposits at Sterkfontein. The Past, Present and Future of Hominid Evolutionary Studies.

